# Supplementary material for: Molecular basis of enzymatic nitrogen-nitrogen formation by a family of zinc-binding cupin enzymes
Source: Nat Commun. 2021 Dec 10;12:7205. doi: 10.1038/s41467-021-27523-x (PMC8664883; doi:10.1038/s41467-021-27523-x)
Supplement: Supplementary file 1 — Supplementary Information [file 41467_2021_27523_MOESM1_ESM.pdf]

Supplementary Information

Molecular basis of enzymatic nitrogen-nitrogen formation by a family of zinc-binding cupin enzymes

Zhao et al.

Supplementary Table 1. Primers used in this study

Primer name	Sequence (5′ ⟶ 3′)	Discriptation
nbtG-BamHI-F	agcagcGGATCCgaaaccctgctggttgttg	Primers for *nbtG* cloning
nbtG-HindIII-R	agcagcAAGCTTtcactgtgctgccagctgacgt	
pyrN-NdeI-F	agcagcCATATGatcgtcagtgagatcccc	Primers for *pyrN* cloning
pyrN-XhoI-R	agcagcCTCGAGtgtcggtgtcggtgtcggtgt	
PyrN-MetRS-NdeI-F	agcagcCATATGgaacaggctgtggaacggcag	Primers for *pyrN-MetRS* cloning
PyrN-MetRS-XhoI-R	agcagcCTCGAGctgcctcccaggtgctgttcg	
PyrN-cupin-NdeI-F	agcagcCATATGatcgtcagtgagatcccc	Primers for *pyrN-cupin* cloning
PyrN-cupin-XhoI-R	agcagcCTCGAGtcactcccaccacatcgagaagaac	
pyrN-K458A-F	GCGttctccaccggccgcaagcac	Primers for point mutation of *pyrN*
pyrN-K458A-R	ctccccgtcgaggaggtagaag	
pyrN-E56A-F	GCggtctgggtgatgctcgacgg	Primers for point mutation of *pyrN*
pyrN-E56A-R	gttgtgctcggcagtcgtctcg	
pyrN-H50A-cir-F	GCGaaccaccacgacctggaggtctgggtgatgc	Primers for point mutation of *pyrN*
pyrN-H50A-cir-R	ccaggtcgtggtggttCGCctcggcagtcgtctcgcccggcc	
pyrN-H52A-F	GCccacgacctggaggtctgggt	Primers for point mutation of *pyrN*
pyrN-H52A-R	gttgtgctcggcagtcgtctcg	
RHS1-cupin-NdeI-F	agcagcCATATGaccacagactcggtcaccc	Primers for *RHS1* cloning
RHS1-cupin-HindIII-R	agcagcAAGCTTtcacggacgccaccagatcgac	
RHS1-cupin-D63A-F	GCAcaccatgaggtgcgcgagatctgg	Primers for point mutation of *RHS1*
RHS1-cupin-D63A-R	ctcggcggtggtgacgccc	
RHS1-cupin-H65A-F	GCGgaggtgcgcgagatctggctc	Primers for point mutation of *RHS1*
RHS1-cupin-H65A-R	gtgatcctcggcggtggtgac	
RHS1-cupin-E69A-cir-F	caccatgaggtgcgcgCgatctggctcgtccagtccggatc	Primers for point mutation of *RHS1*
RHS1-cupin-E69A-cir-R	actggacgagccagatcGcgcgcacctcatggtgatcctc	
RHA1-cupin-D63A-E69A-cir-F	GCAcaccatgaggtgcgcgCgatctggctcgtc	Primers for point mutation of *RHS1*
RHS-cupin-D63A-E69A-cir-R	gcgcacctcatggtgTGcctcggcggtggtgacgccc	
RHS1-cupin-H103A-cir-F	cgagagctatcgacgcGCGcaactgcacaacgacggcgact ctcc	Primers for point mutation of *RHS1*
RHS1-cupin-H103A-cir-R	CGCgcgtcgatagctctcgtagtacagcgtgtcg	
RHS1-cupin-R26A-F	GCAccctcgggcgcgaaactcggaatc	Primers for point mutation of *RHS1*

RHS1-cupin-R26A-R	gttctcgaagtccagttccggcagtc	
RHS1-cupin-S28A-F	GCAggcgcgaaactcggaatcttcgatc	Primers for point mutation of *RHS1*
RHS1-cupin-S28A-R	gggcctgttctcgaagtccagttc	
RHS1-cupin-L32A-F	GCAggaatcttcgatctgccgaagctcgag	Primers for point mutation of *RHS1*
RHS1-cupin-L32A-R	tttcgcgcccgagggcctgttc	
RHS1-cupin-I52A-F	GCAcgtgtgcccgggggcgtcac	Primers for point mutation of *RHS1*
RHS1-cupin-I52A-R	gtgtgccagcgtgaatggcgc	
RHS1-cupin-T60A-F	GCAgccgaggatcaccatgaggtgcg	Primers for point mutation of *RHS1*
RHS1-cupin-T60A-R	ggtggtgacgcccccgggcacacg	
RHS1-cupin-V67A-F	GCAcgcgagatctggctcgtccagtc	Primers for point mutation of *RHS1*
RHS1-cupin-V67A-R	ctcatggtgatcctcggcggtggtg	
RHS1-cupin-W71A-F	GCActcgtccagtccggatcggggatt	Primers for point mutation of *RHS1*
RHS1-cupin-W71A-R	gatctcgcgcacctcatggtgatc	
RHS1-cupin-W119A-F	GCAtggcgtccgtgaacgccaggccgag	Primers for point mutation of *RHS1*
RHS1-cupin-W119A-R	gatcgacacgatctcgaccggag	
RHS1-MetRS-NdeI-F	agcagcCATATGatgacccagcacctcatcgtgc	Primers for point mutation of *RHS1*
RHS1-MetRS-HindIII-R	gacagcAAGCTTctgacgggatcgctcatcctcg	
Tri28-NdeI-F	agcagcCATATGatcatcagccgttatgacg	Primers for *Tri28* cloning
Tri28-XhoI-R	agcagcCTCGAGagacgatccgccccagggttc	
A0A126Y2P7-NdeI-F	agcagcCATATGatcacgcgtgccttcgacc	Primers for *A0A126Y2P7* cloning
A0A126Y2P7-HindIII-R	agcagcAAGCTTctgttcgaggacgagcaccgcg	
Q9ZBH8-NdeI-F	agcagcCATATGagcggcatccggatccagc	Primers for *Q9ZBH8* cloning
Q9ZBH8-XhoI-R	agcagcCTCGAGtcatcggtcctccccgtgggg	
Q9ZBH7-NdeI-F	agcagcCATATGacaccggccccgcgccgct	Primers for *Q9ZBH7* cloning
Q9ZBH7-XhoI-R	agcagcCTCGAGtgcggtgcggtgcggtcagagg	
RHS1-cupin-E69D-cir-F	caccatgaggtgcgcGATatctggctcgtccagtccggatc	Primers for point mutation of *RHS1*
RHS1-cupin-E69D-cir-R	agatAtcgcgcacctcatggtgatcctcggcggtggtg	Primers for point mutation of *RHS1*
RHS1-cupin-E69N-cir-F	caccatgaggtgcgcAACatctggctcgtccagtccggatc	Primers for point mutation of *RHS1*

RHS1-cupin-E69N-cir-R	agatGTTgcgcacctcatggtgatcctcggcggtggtg	Primers for point mutation of *RHS1*
RHS1-cupin-E69Q-cir-F	caccatgaggtgcgcCAGatctggctcgtccagtccggatc	Primers for point mutation of *RHS1*
RHS1-cupin-E69Q-cir-R	agatctGgcgcacctcatggtgatcctcggcggtggtg	Primers for point mutation of *RHS1*
RHS1-cupin-E69L-cir-F	caccatgaggtgcgcCTGatctggctcgtccagtccggat	Primers for point mutation of *RHS1*
RHA1-cupin-E69L-cir-R	agatcAGgcgcacctcatggtgatcctcggcggtggtg	
RHA1-LysOH-MetRS-NdeI-F	AGCAGCCATatgttggaacaccttgatctggttgg	Construction of pTips-QC1-LysOH-MetRS-RHS1
RHA1-LysOH-MetRS-HindIII-R	AGCAGCAAGCTTgatcgctcatcctcggcctccac	Construction of pTips-QC1-LysOH-MetRS-RHS1
RHS1-HindIII-F	AGCAGCAAGCTTcgaacccattgaaggagttagc	Construction of pTips-QC1-LysOH-MetRS-RHS1
RHS1-HindIII-R	AGCAGCAAGCTTgaagaacatcacgctcaagctcg	Construction of pTips-QC1-LysOH-MetRS-RHS1

Supplementary Fig. 1

a

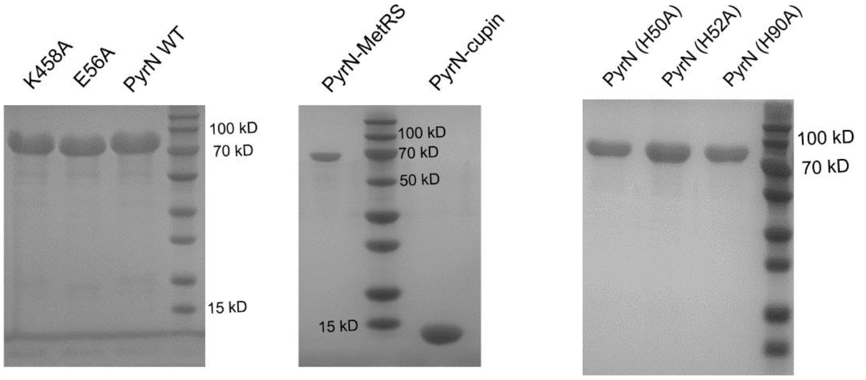

b

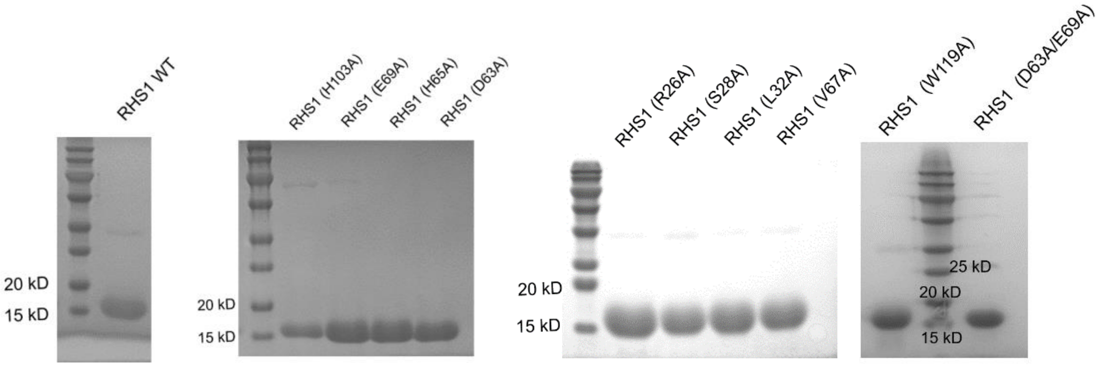

c

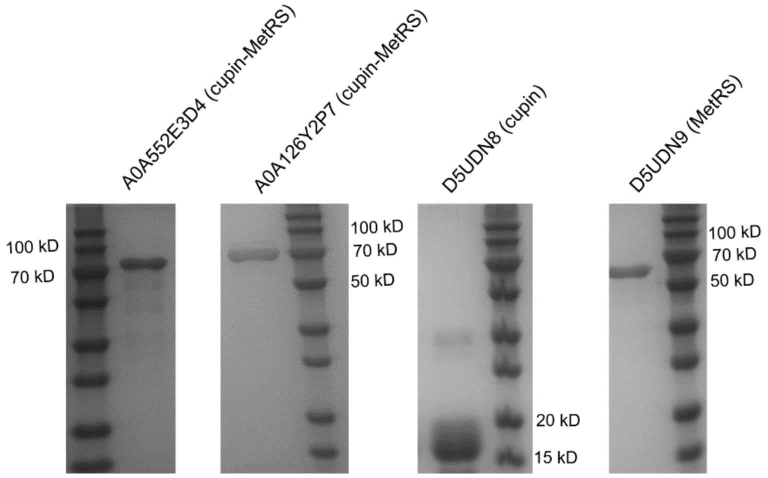

Supplementary Fig. 1 SDS-PAGE analysis of enzymes used in this study. (a) PyrN and its variants or truncated versions. The molecular weight for each protein: *N*-His$_6$-PyrN (74.96 kD), *N*-His$_6$-MetRS domain (61.39 kD), *N*-His$_6$-cupin domain (14.00 kD). (**b**) RHS1 and its variants (see below). The size of *N*-His$_6$-RHS1: 15.55 kD. (**c**) Enzymes obtained from database mining and used for substrate specificity test (see below). Note: all the above experiments were repeated three times independently, and similar results were obtained.

Supplementary Fig. 2

a

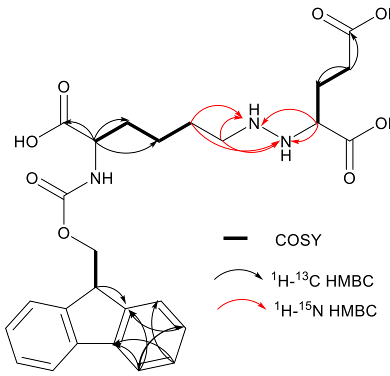

NMR data of Compound **1**-Fmoc[a]					
NO.	δ_H	δ_C/δ_N	NO.	δ_H	δ_C
1	-	174.63	12	2.39,2.29 (2H,brs)	31.88
2	3.53 (1H,brs)	55.94	13	-	175.54
3	1.73 (2H,brs)	31.76	15	-	158.68
4	1.22 (2H,brs)	23.47	16	4.53 (2H,brs)	68.47
5	1.28 (2H,brs)	30.75	17	4.23 (1H,brs)	48.55
6	3.16 (2H,m)	50.55	18,18’	-	145.30
N-7	-	122.90	19,19’	7.59 (2H,brs)	125.80
N-8	-	89.68	20,20’	7.30 (2H,m)	128.25
9	3.37 (1H,brs)	65.03	21,21’	7.39 (2H,m)	128.82
10	-	179.31	22,22’	7.80 (2H,m)	121.04
11	1.80 (2H,brs)	28.10	23,23’	-	142.74

a ^{1}H (600 MHz) and ^{13}C (150 MHz) NMR Data in MeOD.

b

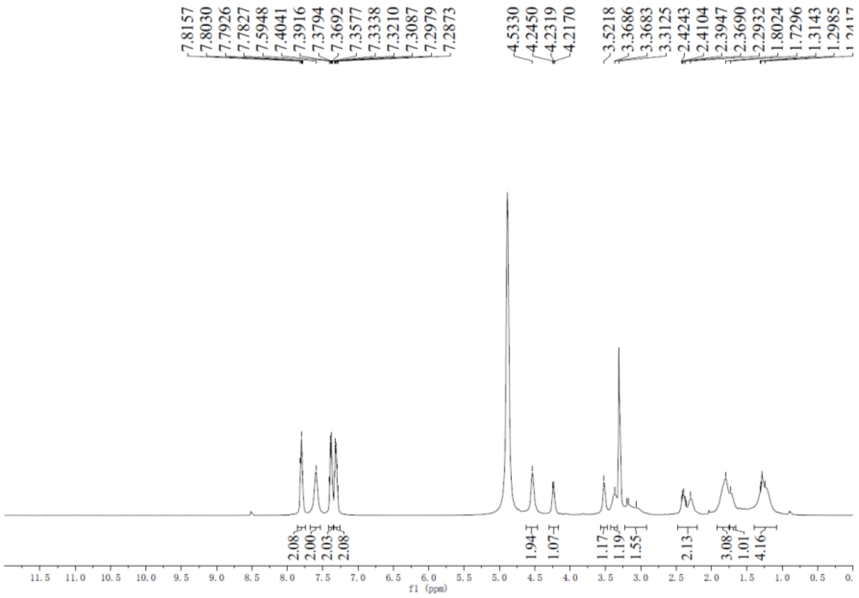

c

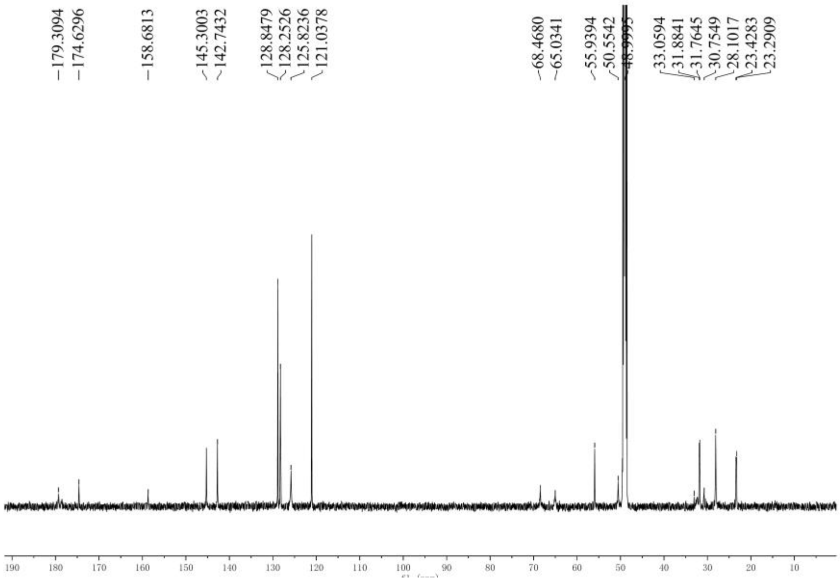
179.3094
174.6296
158.6813
145.3003
142.7432
128.8479
128.2526
125.8236
121.0378
68.4680
65.0341
55.9394
50.5542
33.0594
31.8841
31.7645
30.7549
28.1017
23.4283
23.2909
190 180 170 160 150 140 130 120 110 100 90 80 70 60 50 40 30 20 10

d

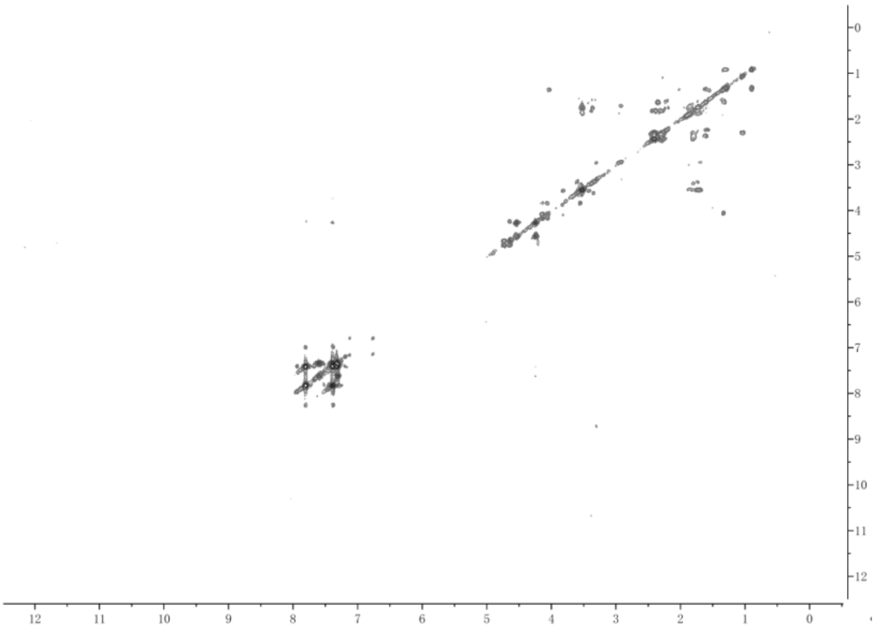
12 11 10 9 8 7 6 5 4 3 2 1 0
0 1 2 3 4 5 6 7 8 9 10 11 12

e

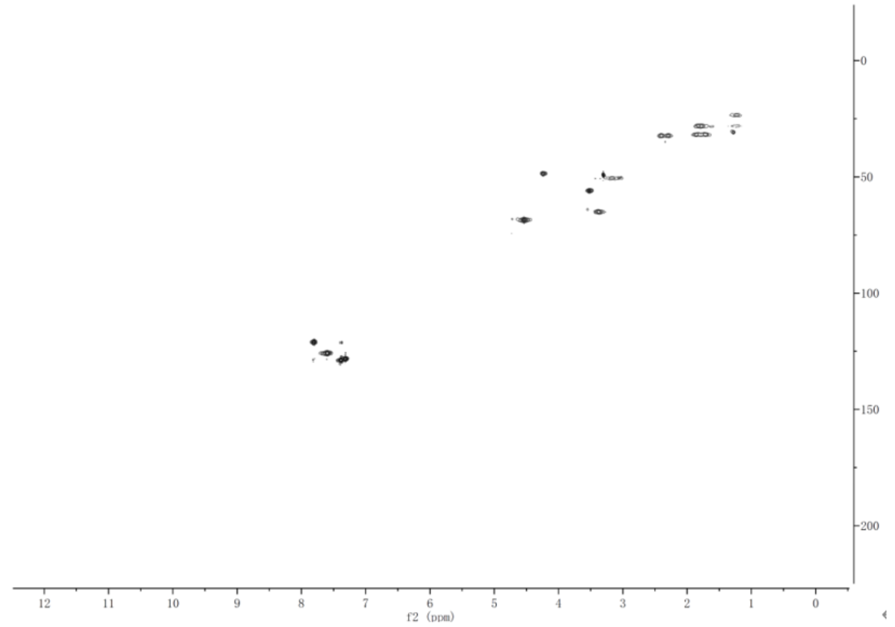
12
11
10
9
8
7
6
5
4
3
2
1
0
f2 (ppm)
0
50
100
150
200

f

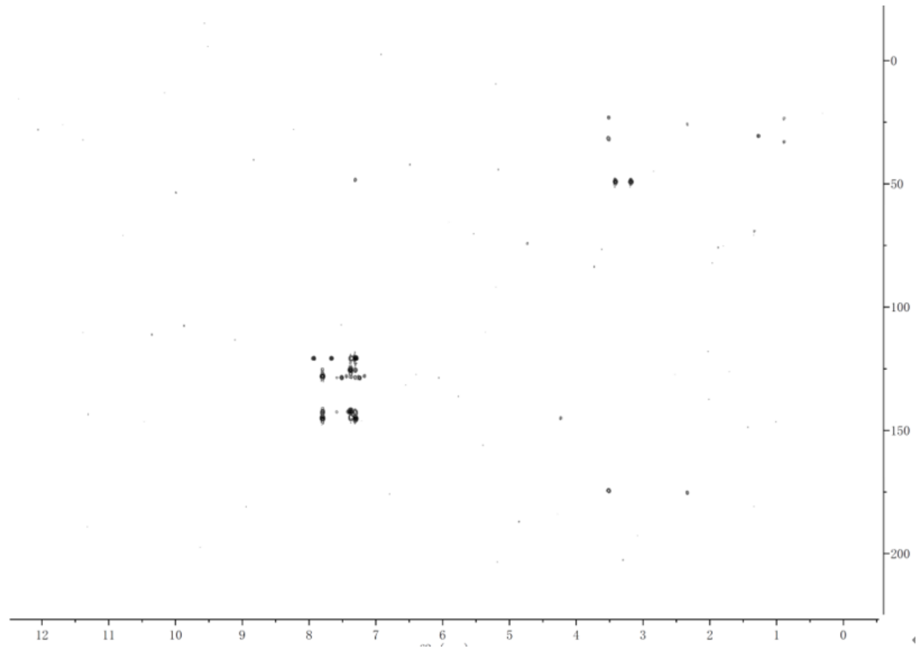
12
11
10
9
8
7
6
5
4
3
2
1
0
0
50
100
150
200

g

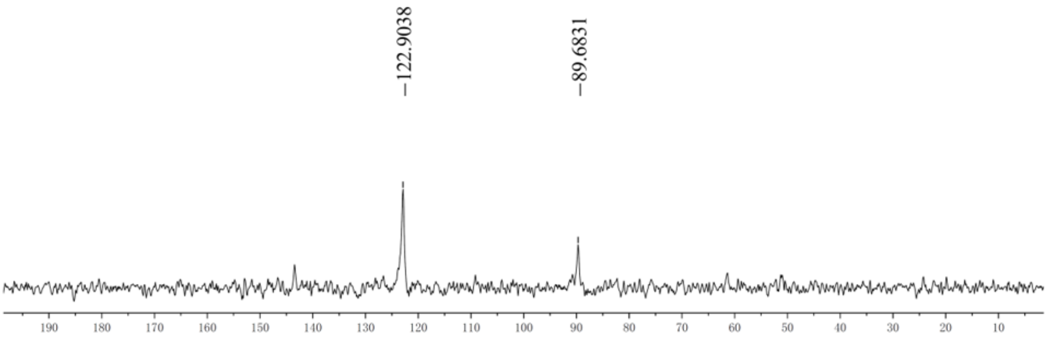

h

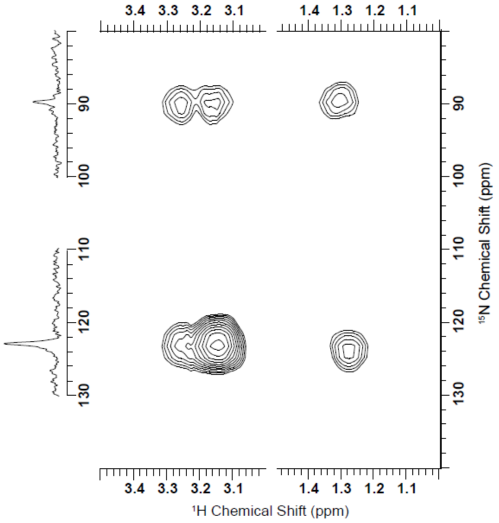

Supplementary Fig. 2 Structural assignment of isolated Fmoc-**1** based on extensive NMR analysis. (**a**) Structural assignment of isolated Fmoc-**1**. (**b**) ^{1}H NMR spectrum of Fmoc-**1**. (**c**) ^{13}C NMR spectrum of Fmoc-**1**. (**d**) ^{1}H-^{1}H-COSY NMR spectrum of Fmoc-[$^{15}N_2$]-**1**. (**e**) HSQC NMR spectrum of Fmoc-**1**. (**f**) ^{1}H-^{13}C-HMBC NMR spectrum of Fmoc-**1**. (**g**) ^{15}N NMR spectrum of Fmoc-**1**. (**h**) ^{1}H-^{15}N-HMBC NMR spectrum of Fmoc-[$^{15}N_2$]-**1**.

Supplementary Fig. 3

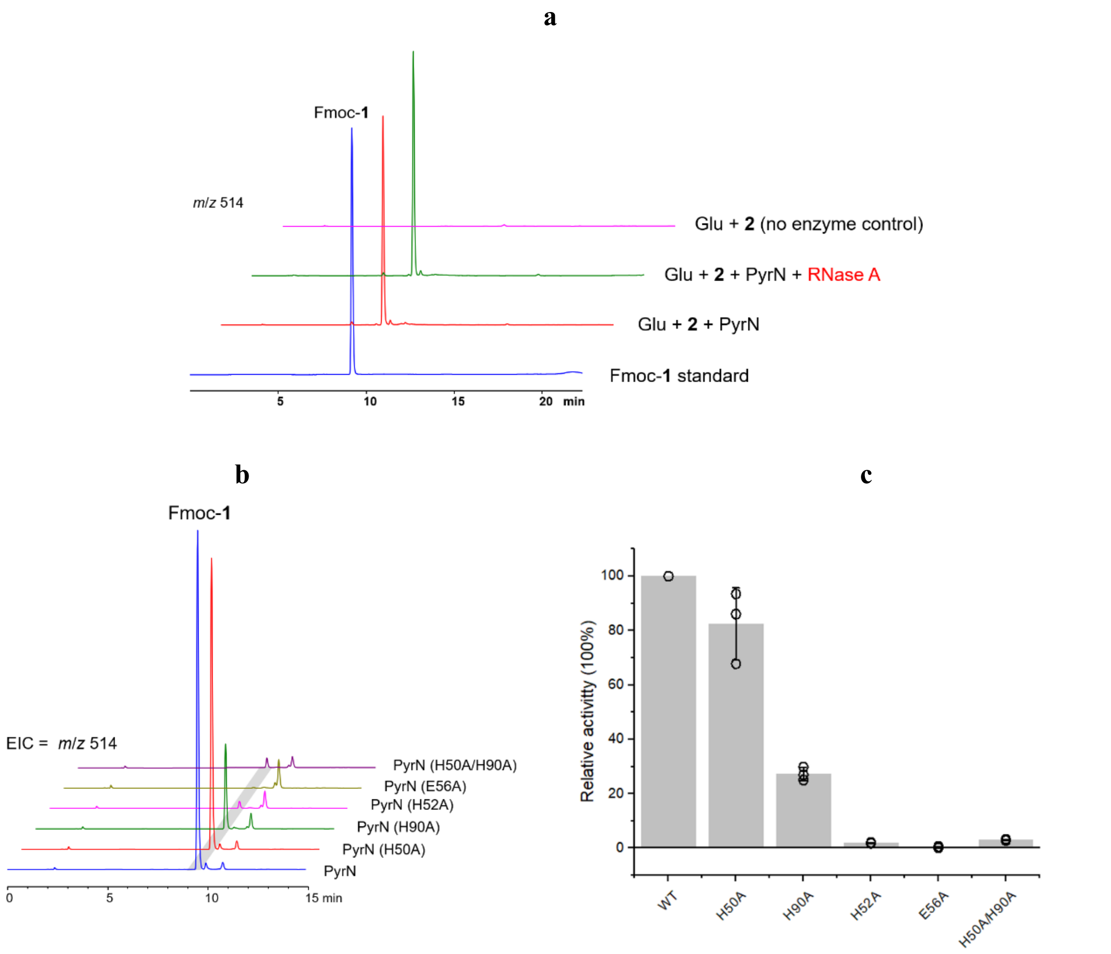

Supplementary Fig. 3 In vitro biochemical assays for PyrN and its variants. (**a**) PyrN-catalyzed tRNA-independent formation of **1**. Extracted ion chromatograms (EIC = *m*/*z* 514, $[M+H]^+$ ion for Fmoc-**1**) from the LC-MS analysis of PyrN reaction mixtures were displayed. The result reveals that addition of RNase A has no effect on the production of **1**. (**b**) Extract ion chromatograms (EIC = *m*/*z* 514, $[M+H]^+$ ion for Fmoc-**1**) from the LC-MS analysis of the reaction mixtures of PyrN and its variants. (**c**) Relative activities of PyrN variants as compared to the wild type (WT) PyrN. Three independent tests were taken. Values are means of three independent experiments ± SD (n=3).

Supplementary Fig. 4

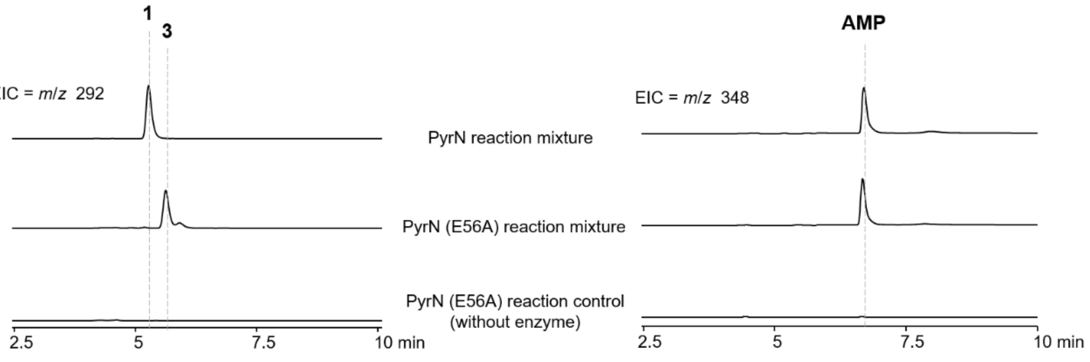

Supplementary Fig. 4 Extract ion chromatograms (EIC = *m*/*z* 514, $[M+H]^+$ for **1** and **3**, and *m*/*z* 348, $[M+H]^+$ for AMP) from the LC-MS analysis of the reaction mixtures containing PyrN or PyrN (E56A), which reveals the production of AMP in both reaction mixtures.

Supplementary Fig. 5

a

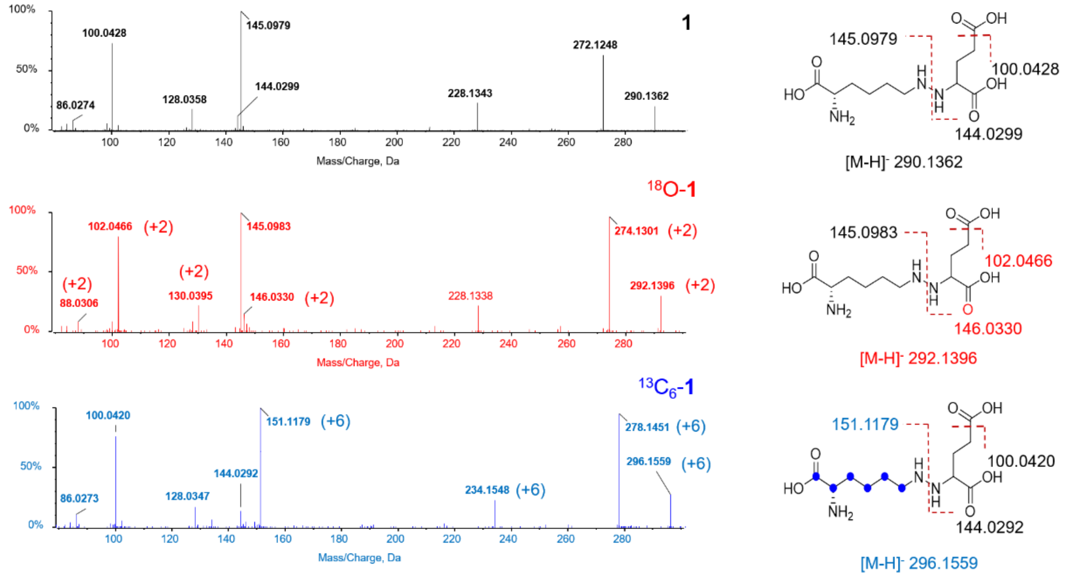

b

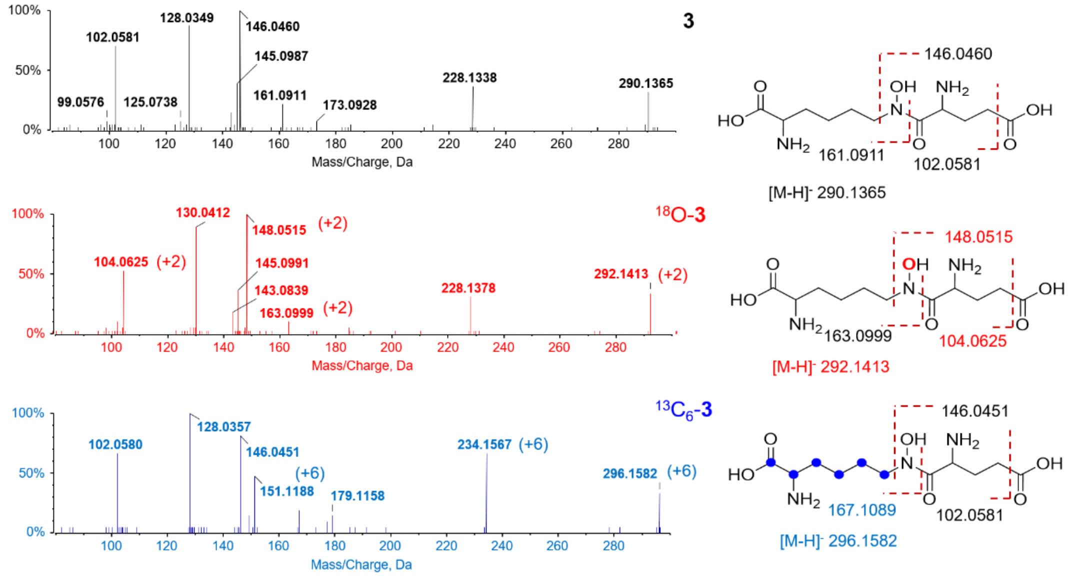

Supplementary Fig. 5 LC-HR-MS/MS analysis of **1** (**a**) and **3** (**b**) in the reaction mixtures containing ^{18}O-labeled or $^{13}C_6$-labeled substrate L-N^6-^{18}OH-Lys and L-N^6-OH-$^{13}C_6$-Lys. Selected diagnostic fragment signals are indicated in the chemical structures of **1** and **3**.

Supplementary Fig. 6

a

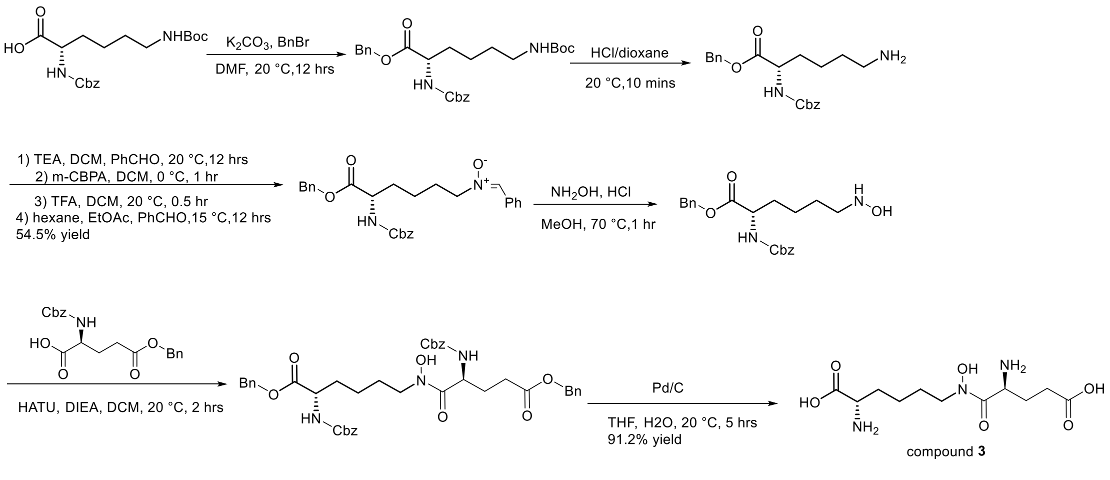

b

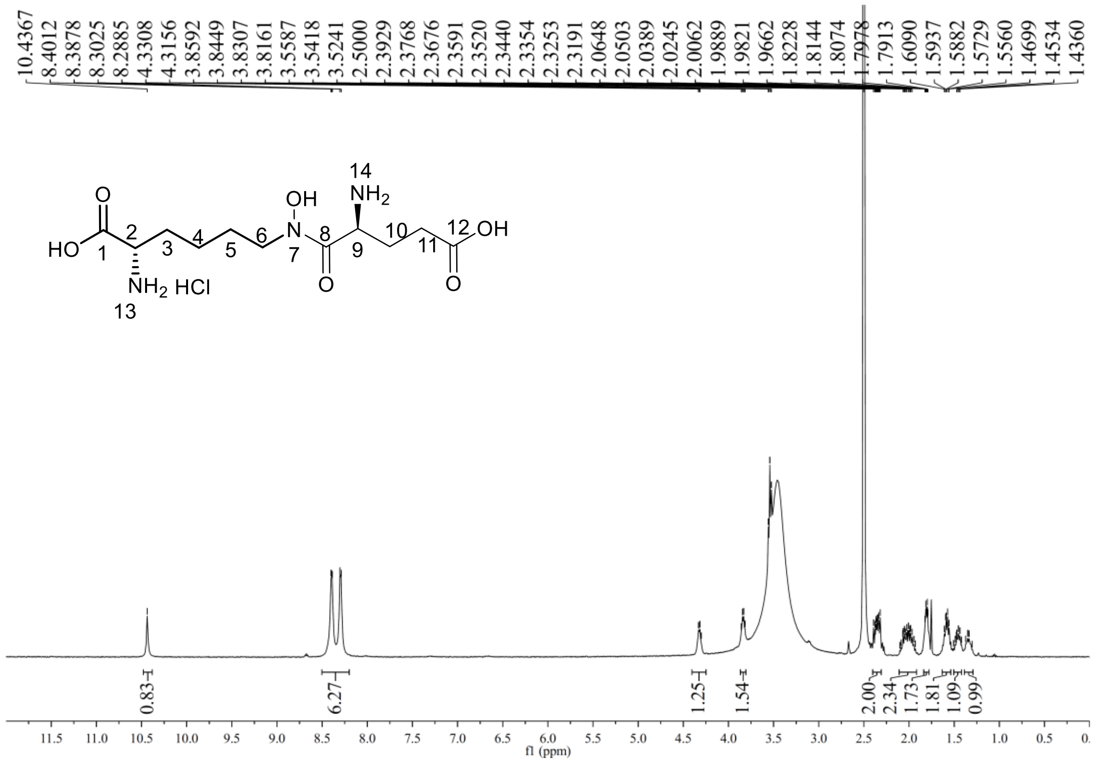

c

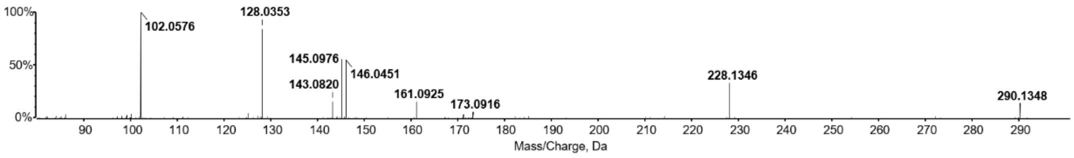

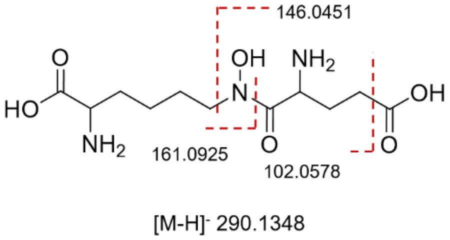

Supplementary Fig. 6 Chemical synthesis route (**a**), ^{1}H NMR spectrum (DMSO-d^{6}, 400 MHz) (**b**) and LC-HR-MS/MS analysis (**c**) of **3** synthetic standard. ^{1}H NMR data for synthetic **3**: ^{1}H-NMR (400 MHz, DMSO-d6) δ10.44 (H, s, N7-OH); 8.40,8.30 (6H, s, NH-13,14); 4.31 (H, m, H-2); 3.84 (H, m, H-9); 3.54 (1H, s, H6); 2.35 (2H, m, H-11); 2.02 (2H, m, H-10); 1.80 (2H, m, H-3); 1.58 (2H, m, H-5); 1.47,1.34 (2H, m, H-4)

Supplementary Fig. 7

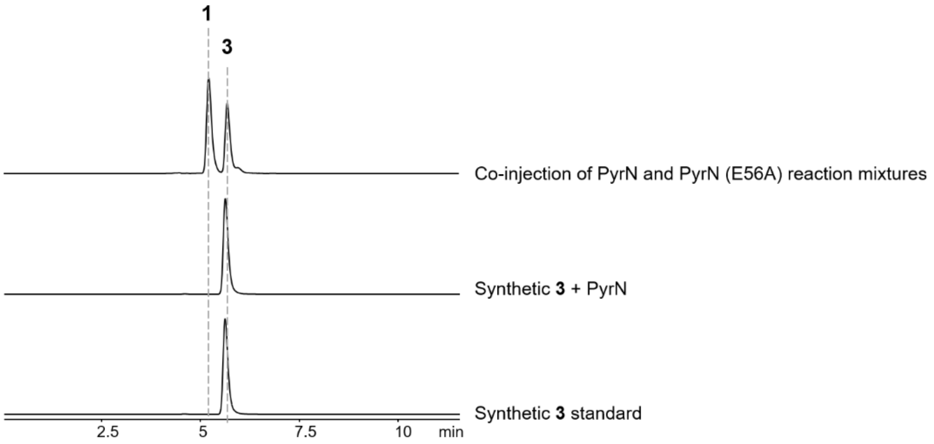

Supplementary Fig. **7** LC-MS analysis (EIC = *m*/*z* 292, [M+H]$^+$ ion for **1** and **3**) of the *in vitro* reaction mixture of PyrN with **3** revealed no reaction of **3** with PyrN.

Supplementary Fig. 8

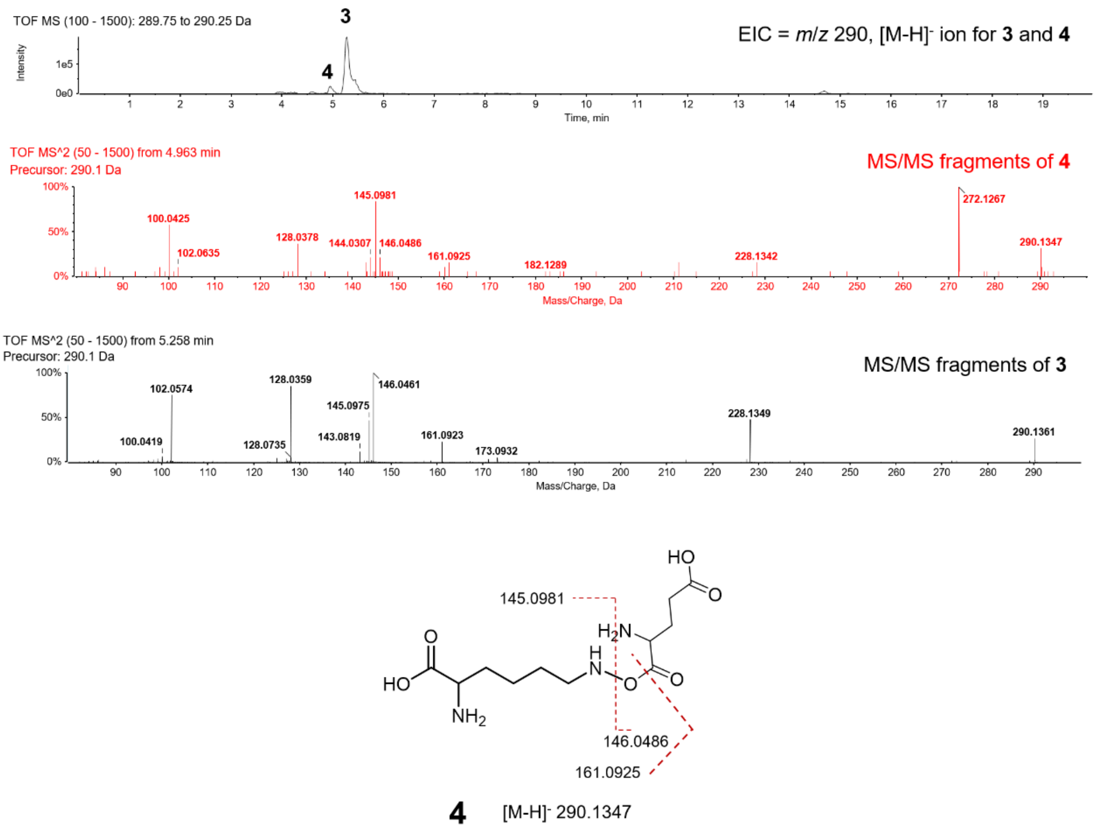

Supplementary Fig. 8 LC-HR-MS/MS analysis (under negative mode) of **4** and **3** in the reaction mixture of PyrN (E56A) after quickly quenched by ultracentrifugation. Selected diagnostic fragment signals are indicated in the chemical structures of **4**.

Supplementary Fig. 9

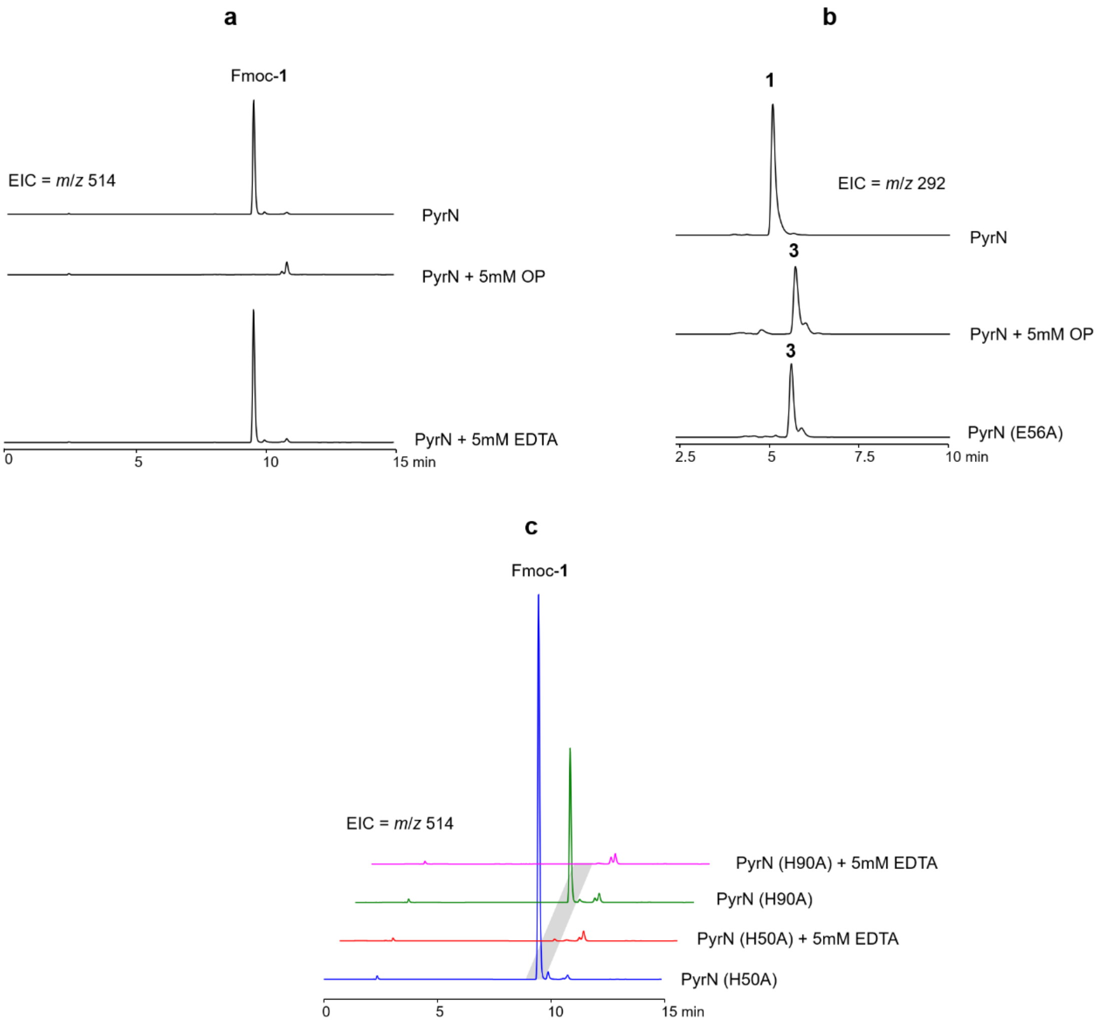

Supplementary Fig. 9 Inhibition of the reactions of PyrN and its variants by metal-chelating agents 1,10-phenanthroline (OP) and ethylenediaminetetraacetic acid (EDTA). (**a**) LC-MS analysis (EIC=*m*/*z* 514, $[M+H]^+$ ion for Fmoc-**1**) of PyrN reaction mixtures containing OP or EDTA, after pre-column Fmoc-Cl derivatization. (**b**) LC-MS analysis (EIC=*m*/*z* 292, $[M+H]^+$ ion for **1**) of PyrN reaction mixtures containing OP or EDTA without derivatization. (**c**) LC-MS analysis (EIC=*m*/*z* 514, $[M+H]^+$ ion for Fmoc-**1**) of the reaction mixtures of PyrN (H90A) or PyrN (H50A) containing EDTA after pre-column Fmoc-Cl derivatization.

Supplementary Fig. 10

a

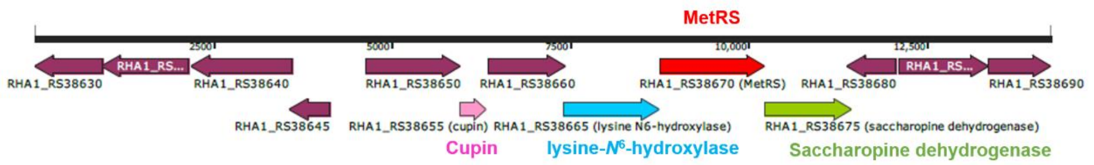

b

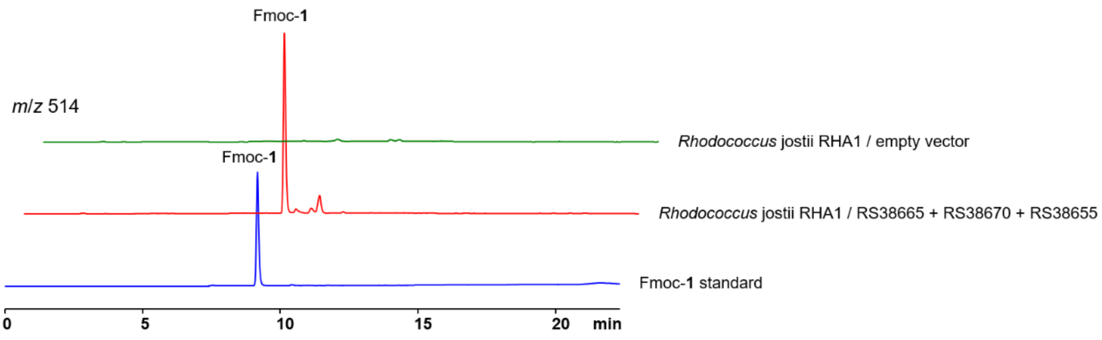

Supplementary Fig. 10 Identification and characterization of a subcluster harboring the PyrN homolog in *Rhodococcus jostii* strain RHA1. (**a**) A putative BGC from *Rhodococcus jostii* strain RHA1 containing homologues of *pyrM* (lysine N^6-hydroxylase), *pyrL* (saccharopine dehydrogenase) and the naturally occurring standalone cupin and MetRS-like protein encoding genes. (**b**) Identification of Fmoc-**1** as the product from the *Rhodococcus* strain overexpressing lysine N^6-hydroxylase (RS38665), MetRS-like (RS38670) and cupin (RS38655) proteins. Note: these three genes were cloned into the *Rhodococcus* replicative vector pTip-QC1 and expressed in *Rhodococcus jostii* strain RHA1, under the control of thiostrepton-inducible promoter ptipA. Fmoc-**1** could be successfully detected in the resulting engineered strain, whereas no production of Fmoc-**1** was found in the control strain that only carries the empty pTip-QC1 vector, suggesting that these genes are cryptic in the wild type *Rhodococcus jostii* RHA1 strain.

Supplementary Fig. 11

a

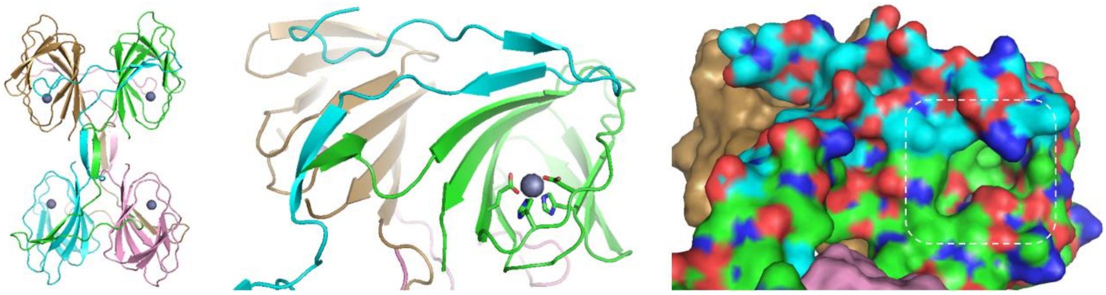

b

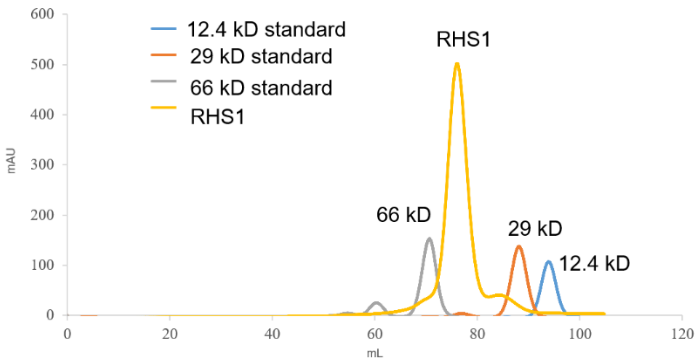

Supplementary Fig. 11 The crystal structure of RHS1 (reproduced from PDB: 5UQP) (**a**) and FPLC analysis of RHS1 (**b**). (**a**) The crystal structure of RHS1. A putative solvent-accessible active site, where the zinc ion is bound, is indicated using a white box (right panel). (**b**) FPLC analysis of RHS1 using a HiLoad 16/600 Superdex 200pg column. The result demonstrated that tag-less RHS1 forms a homotetramer in solution. Note: the molecular weight of a RHS1 monomer (with His_6-tag removed by TEV digestion) is 13.6 kD.

Supplementary Fig. 12

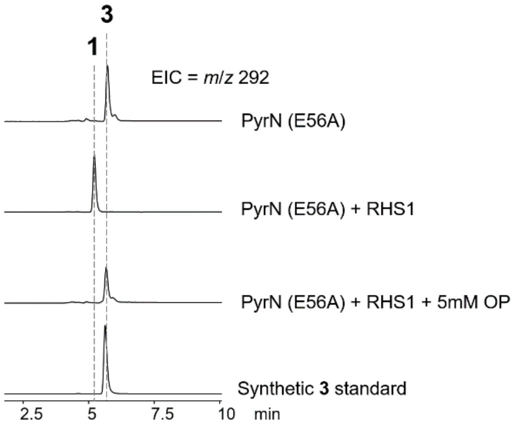

Supplementary Fig. 12 Inhibition of RHS1-catalyzed reaction by the metal-chelating agent 1,10-phenanthroline (OP).

Supplementary Fig. 13

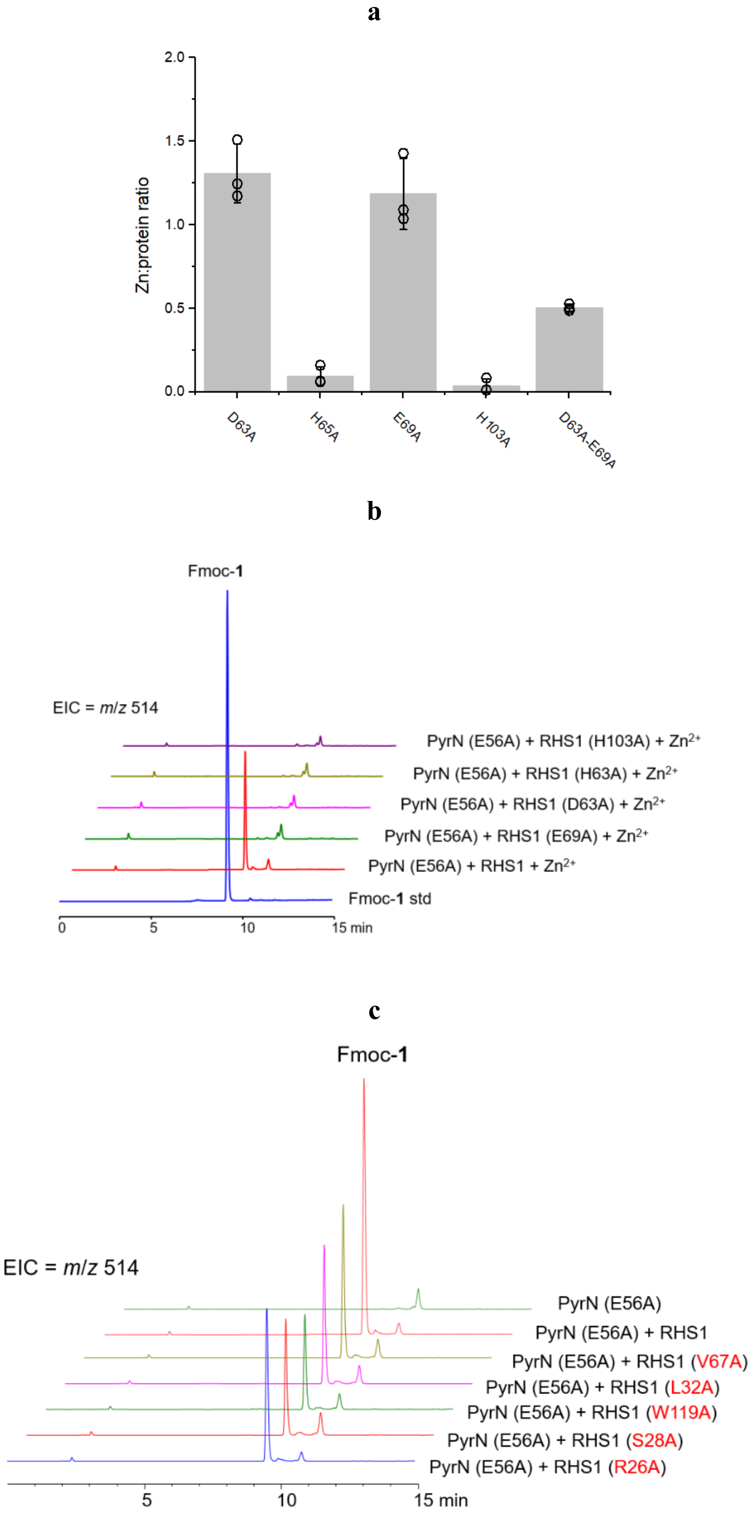

Supplementary Fig. 13 Characterization of RHS1 variants. (**a**) ICP-MS analysis reveals the zinc contents of RHS1 variants. Three independent tests were taken. Values are means of three independent experiments ± SD (n=3). (**b**) LC-MS analysis of the reaction mixtures of RHS1 variants in the presence of zinc ion at the concentration of 100 μM. (**c**) LC-MS analysis (EIC=*m*/*z* 514, $[M+H]^+$ ion for Fmoc-**1**) of the *in vitro* reaction mixtures of RHS1 variants (in coupled reactions with PyrN (E56A), which provided highly unstable **4** *in situ*).

Supplementary Fig 14.

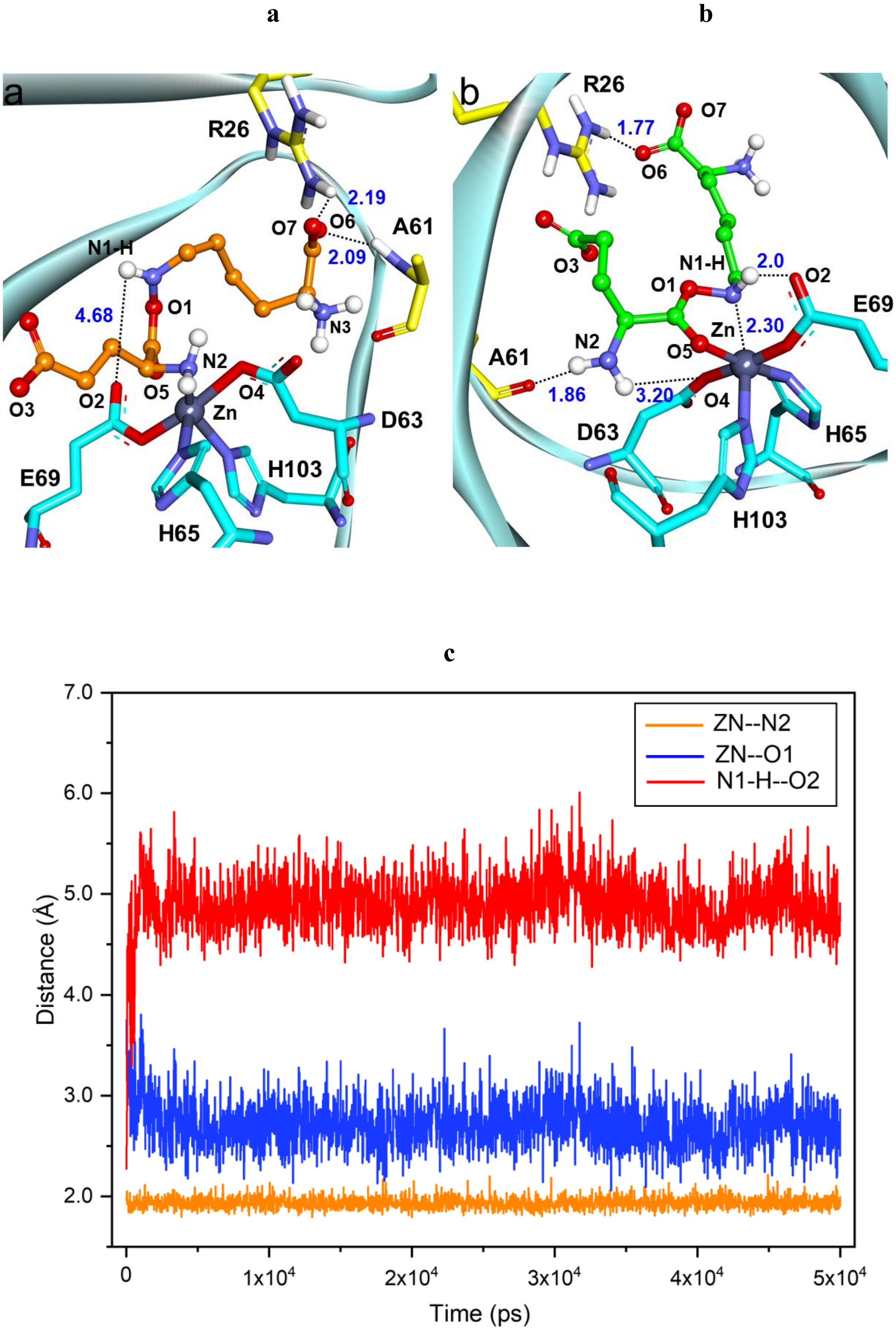

d

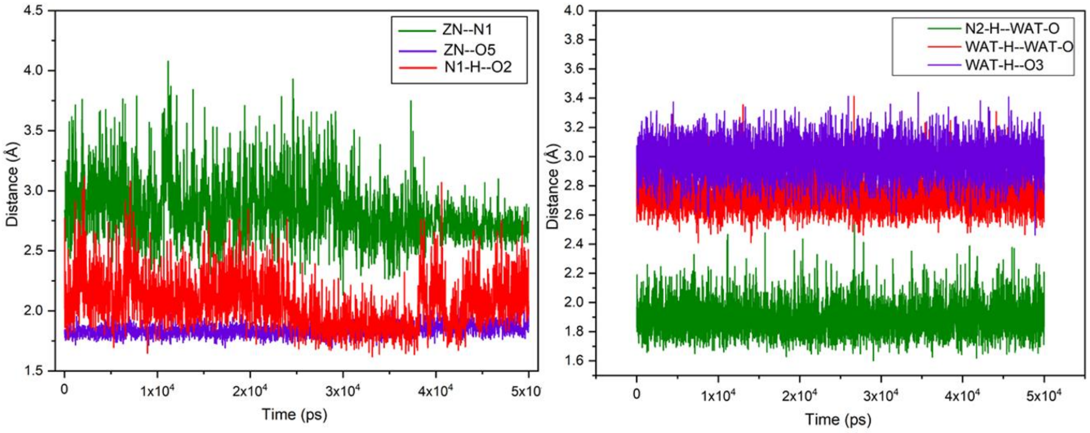

Supplementary Fig. 14 Two representative docking conformations of **4** in the zinc-binding cupin enzyme RHS1 obtained by molecular docking. (**a**) Conformation-1. In this conformation, N2 of amino group is coordinated to zinc, while the carboxyl group of **4** forms a salt-bridge with Arg26. (**b**) Conformation-2. In this conformation, O5 and N1 of **4** are coordinated to zinc. Meanwhile, the imino group (N1-H) forms an H-bond with the O2 of carboxyl group in Glu69. Another two H-bonds can also be identified, including the one between O6 and Arg26, the one between N2 and Ala61. The ball-and-stick model exposes docking conformations of substrate, conformation-1 is colored by orange, conformation-2 is colored by green. The key amino acid residues around zinc have been exhibited in cyan and yellow stick model. (**c**) and (**d**) Molecular dynamics (MD) simulations showed that both conformation-1 and conformation-2 are stable. (**c**) The fluctuation of key distance during the 50ns MD simulation of conformation-1 The distance between zinc and N2 in amino group (orange), zinc and O1 atom (blue), and O2 of carboxyl (-COO^-) group in Glu69 and imine (-N1-H) group (red) during the MD simulation of conformation-1. The atom numbering can be found in (**a**). (**d**) The fluctuation of key distance during the 50ns MD simulation of conformation-2. Left panel: the distance between zinc and imine (-N1-H) group (green), zinc and O5 of carbonyl (-C=O) group (purple), and O2 of carboxyl (-COO^-) group in Glu69 and imine (-N1-H) group (red) during the MD simulation. Right panel: the distances of the essential groups (-N2-H_3 and -COO^- groups in main text Fig. 5 and the following Supplementary Fig. 18) of substrate and two water molecules during the 50ns MD simulation. The two water molecules can make stable H-bonds with the H atom of -N2-H_3 and O3 atom of -COO^- in the substrate. The atom numbering can be found in (**b**).

Supplementary Fig. 15

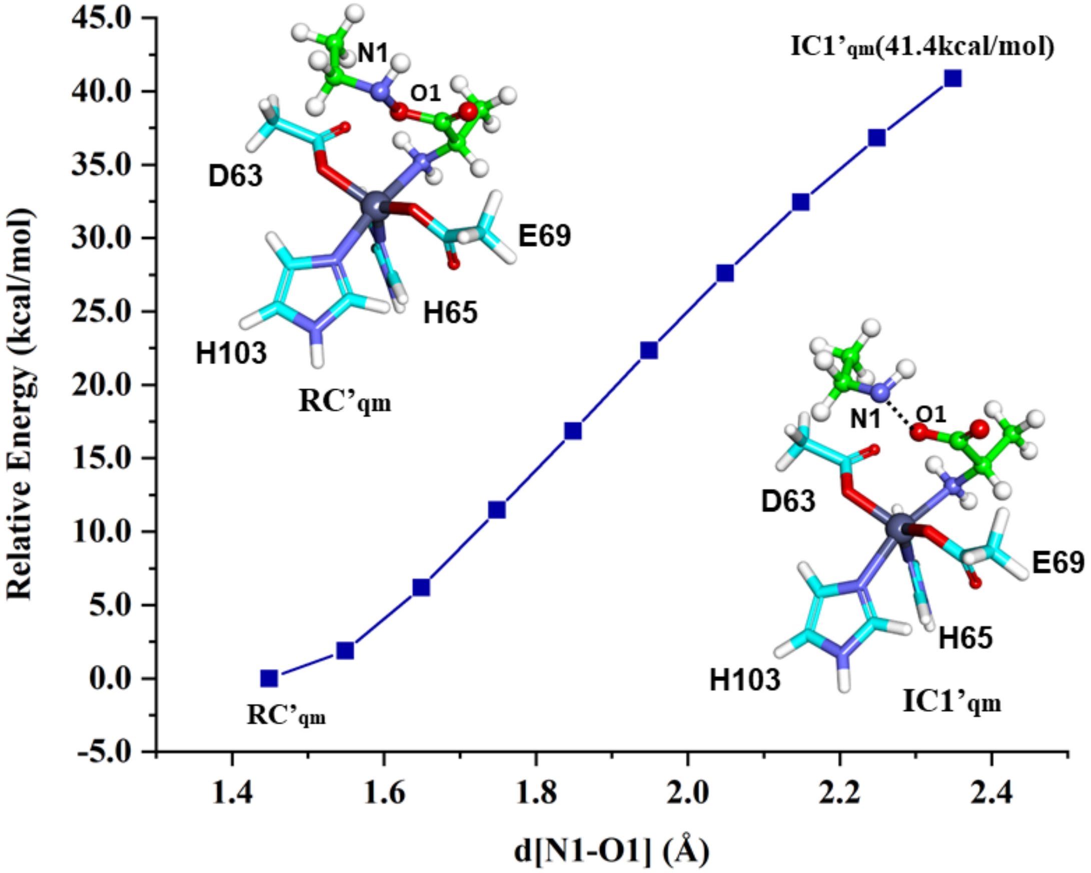

Supplementary Fig. 15 QM calculated energy profile for the N1-O1 cleavage in Conformation-1. The reaction coordinate is defined as the distance between N1 and O1 in substrate. **RC** = reactant complex, **IC** = intermediate complex.

Supplementary Fig. 16

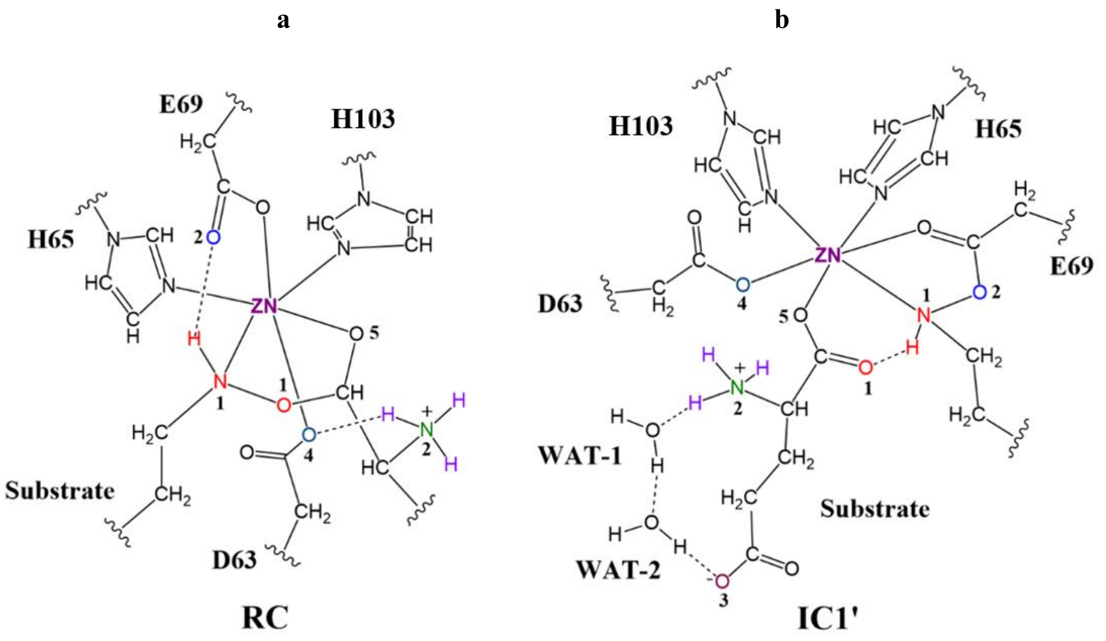

Supplementary Fig. 16 Two QM regions used in QM/MM calculations. (**a**) The QM region of RC used for the initial N1-O1 cleavage (RC→IC1); (**b**) The QM region of IC1' used for all subsequent reaction steps, including the second step of the proton transfer from N2-H_3 to O3 assisted by two bridging waters.

Supplementary Fig. 17

a

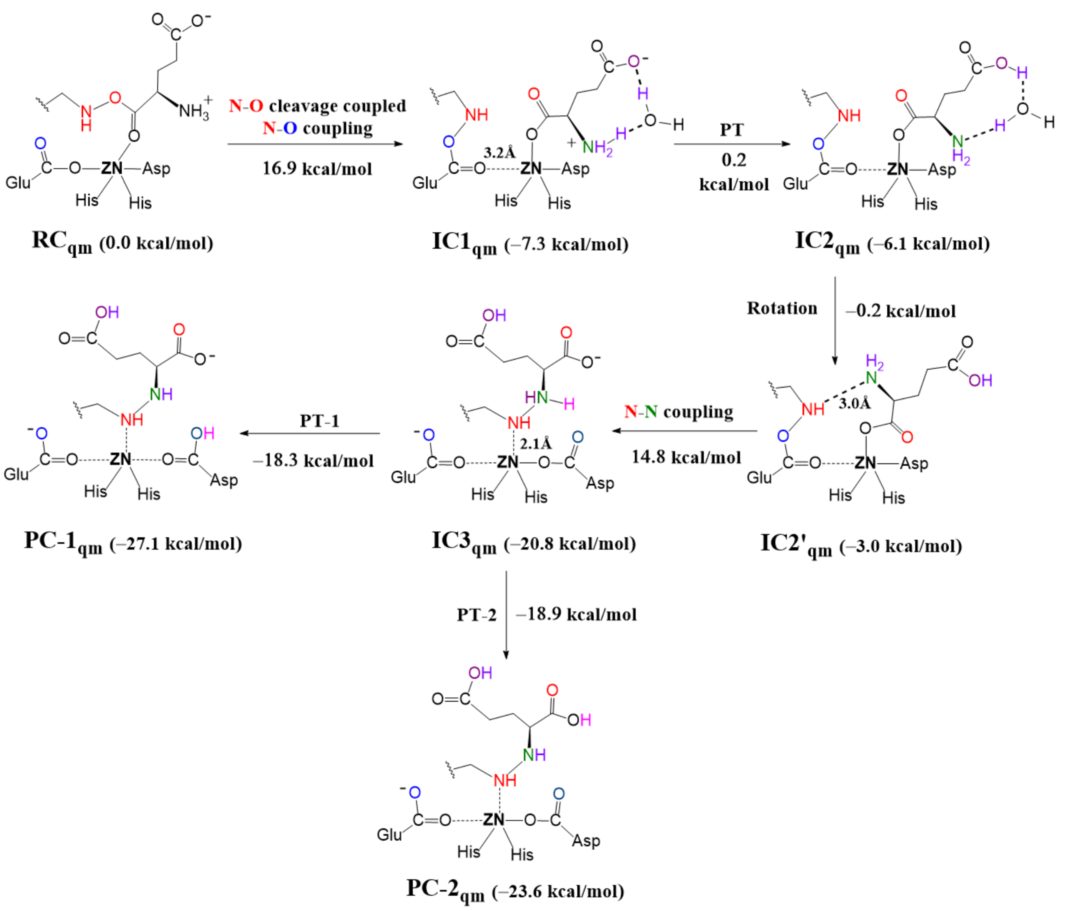

b

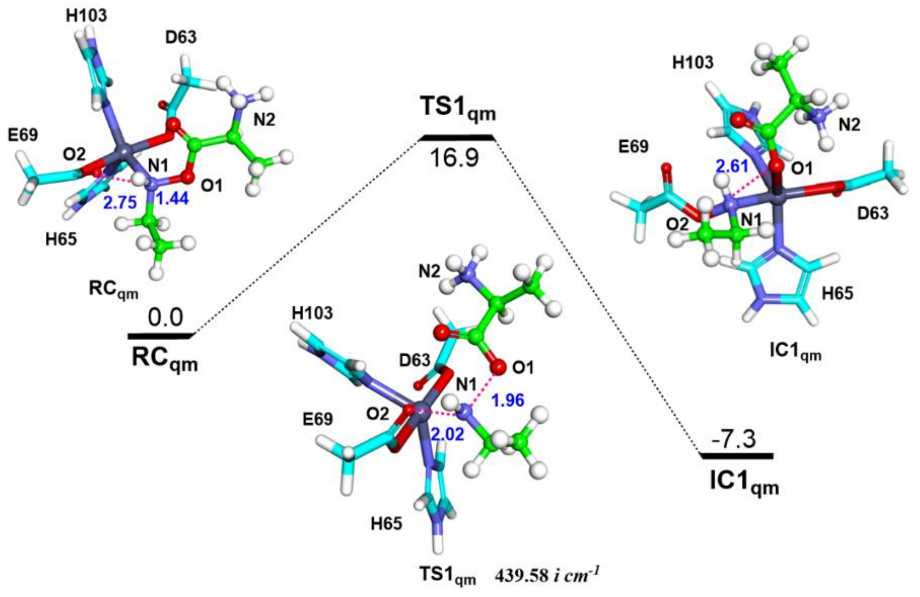

c

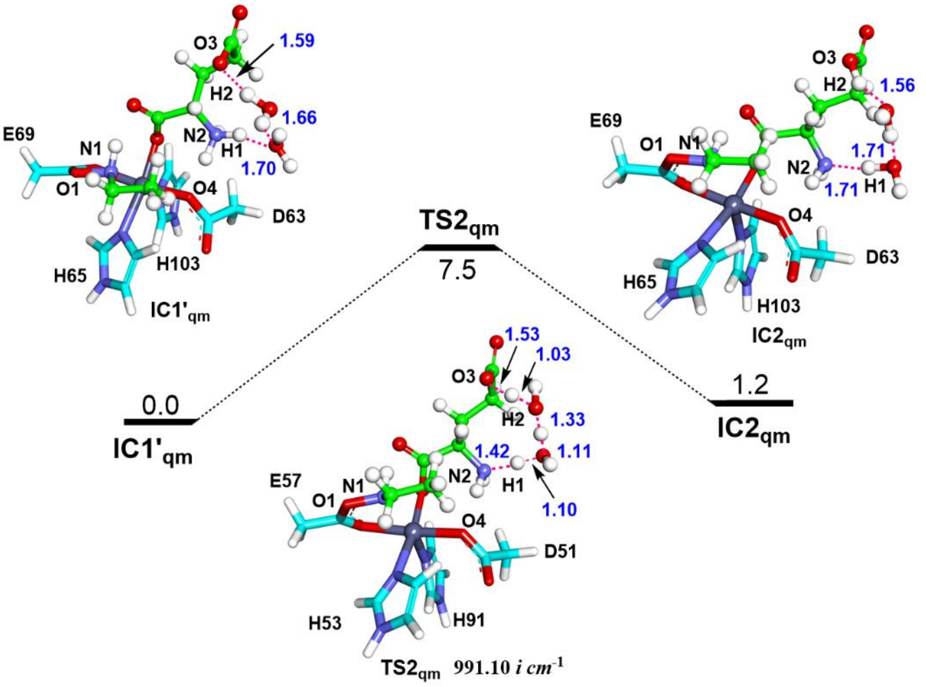

d

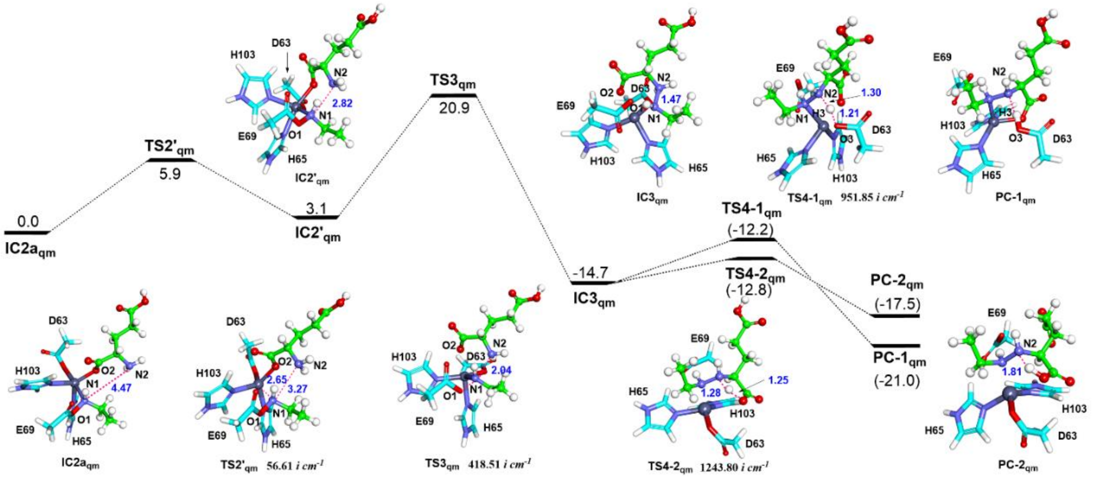

Supplementary Fig. 17 QM calculated free energy profile for the catalytic process of RHS1-catalyzed N-N bond formation. **RC** = reactant complex, **TS** = transition state, **IC** = intermediate complex, **PC** = product complex. (**a**) QM calculated mechanism (free energy) for the N-N bond formation. (**b**) QM calculated free energy profile for the N1-O1 cleavage coupled with the N1-O2 bond formation between the cleaved N1-containing fragment and the carboxyl group of Glu69. (**c**) QM calculated free energy profile for the two organized waters-mediated proton transfer from N2-H_3 to O3 of carboxyl group in the substrate. (**d**) QM calculated free energy profile for the following catalytic process from the amino (-NH_2) group containing intermediate ($IC2a_{qm}$) to the N-N containing product **1** ($PC\text{-}1_{qm}$/$PC\text{-}2_{qm}$) in the presence of the zinc-binding cupin enzyme RHS1.

Starting from conformation-2, our above QM calculations show the N1-O1 cleavage in **4** is coupled with the N1-O2 bond formation between the cleaved N1-containing fragment and the carboxyl group of Glu69. This step experiences a moderate barrier of 16.9 kcal/mol. Thus, Glu69

facilitates the N1-O1 cleavage by making the new N1-O2 bond, through which the cleaved N1-containing fragment can be stabilized in IC1 (RC_{qm} → $IC1_{qm}$, ΔG = −7.3 kcal/mol, as shown in Supplementary Fig. 17b). Next, the proton transfer from NH_3 to O3 of carboxyl group in the substrate, which was assisted by two organized waters (Supplementary Fig. 17c), were found to be quite favorable thermodynamically ($IC1_{qm}$ → $IC2_{qm}$). The step affords the amino (-NH_2) group in $IC2_{qm}$. Since the N1---N2 (shown in Supplementary Fig. 17d) has a long distance of ~ 4.5 Å in $IC2_{qm}$, we first investigated the rotation of N2-H_2 group toward N1-H group. As summarized in Supplementary Fig. 17a and 17d, this step requires a small barrier of 5.9 kcal/mol ($IC2_{qm}$ → IC2' $_{qm}$). In so-formed $IC2'_{qm}$ intermediate, the N2-H_2 group is in close proximity with N1-H group (the distance is ~ 3.0 Å), which is expected to facilitate the N1-N2 bond formation. Starting from $IC2'_{qm}$, the N-N coupling between -NH_2 and -NH leads to the formation of NH-NH_2 containing $IC3_{qm}$ intermediate, which involves a barrier of 17.8 kcal/mol relative to $IC3_{qm}$ ($IC2'_{qm}$ → $IC3_{qm}$). Starting from $IC3_{qm}$, we considered two competing pathways. One involves the proton transfer from NH_2 to the coordinated Asp63, which requires the barriers of 2.5 kcal/mol ($IC3_{qm}$ → PC-1_{qm}). For the alternative pathway involving the proton transfer to adjacent carboxyl group, the reaction experiences a small barrier of 1.9 kcal/mol ($IC3_{qm}$ → PC-2_{qm}). Such QM model calculations are line with the QM/MM results.

Supplementary Fig. 18

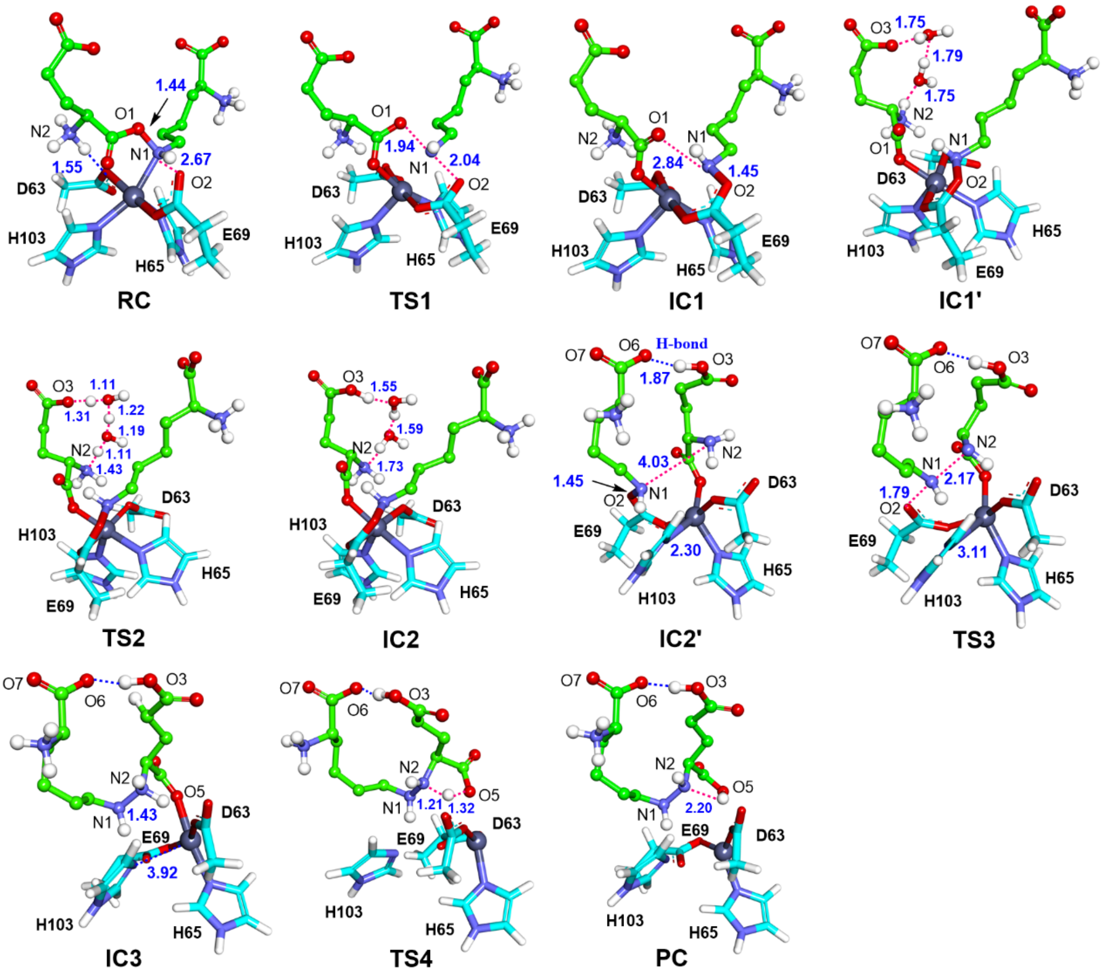

Supplementary Fig. 18 The QM/MM optimized structures of reactant (RC), intermediates (ICs), transition states (TSs) and product (PC) during QM/MM calculation.

Supplementary Fig. 19

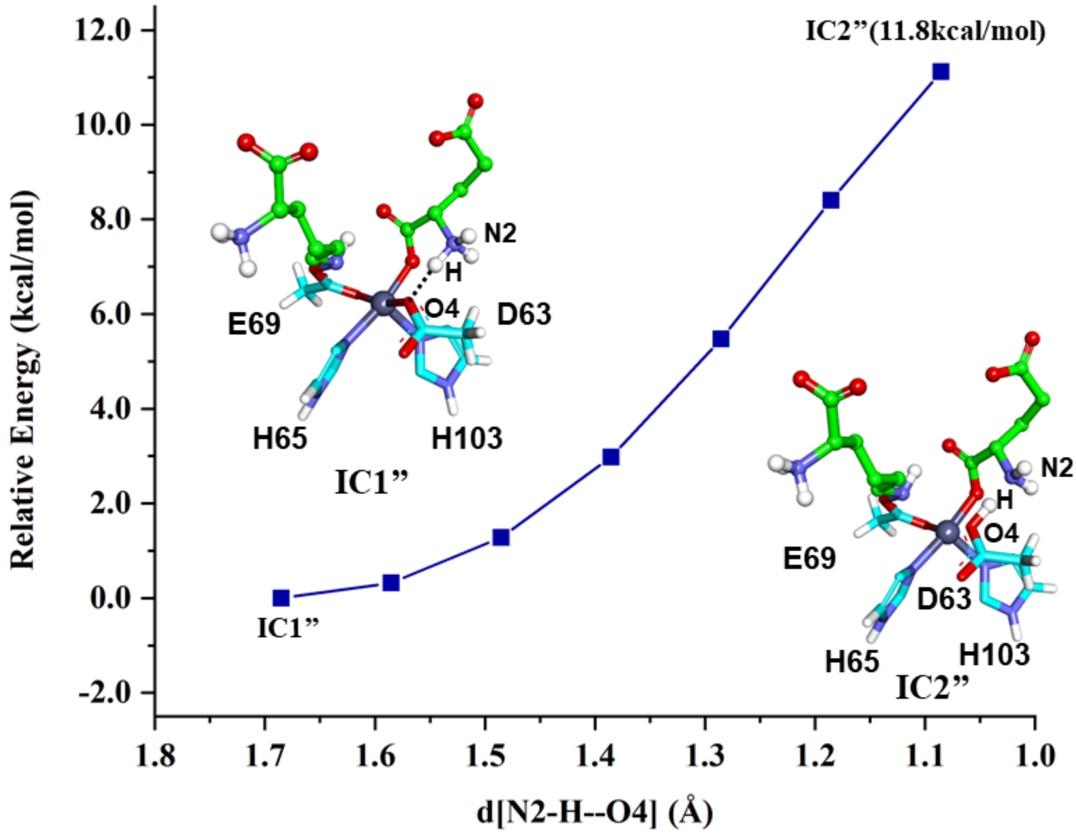

Supplementary Fig. 19 QM/MM calculated energy profile for the proton transformation from N2-H_3 to O4 of carboxyl group in Asp63. The reaction coordinate is defined as the distance between H and O4 in substrate. IC = intermediate complex.

Supplementary Fig. 20

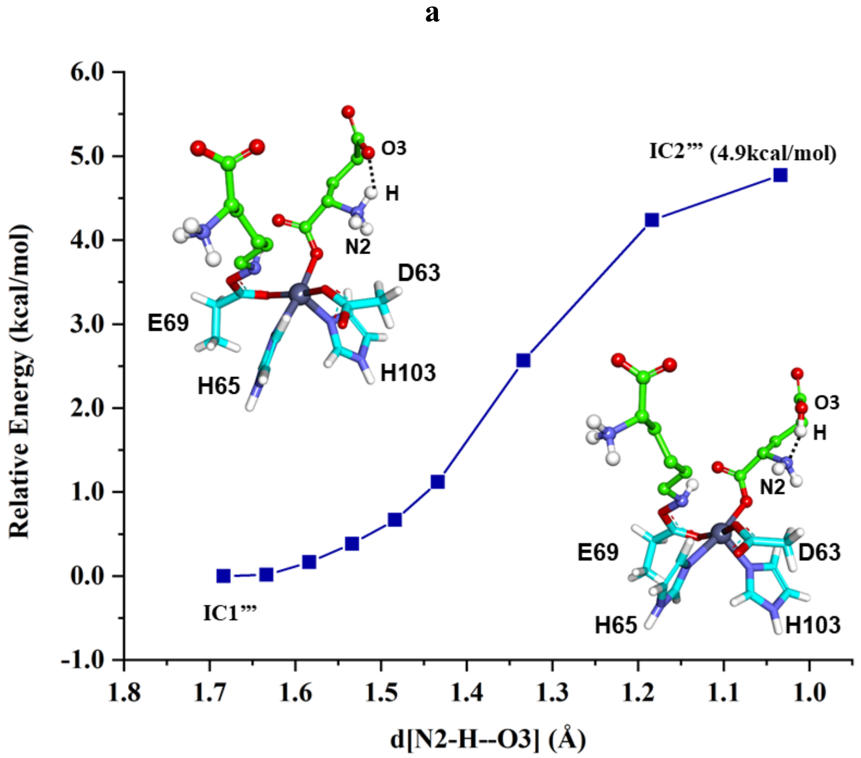

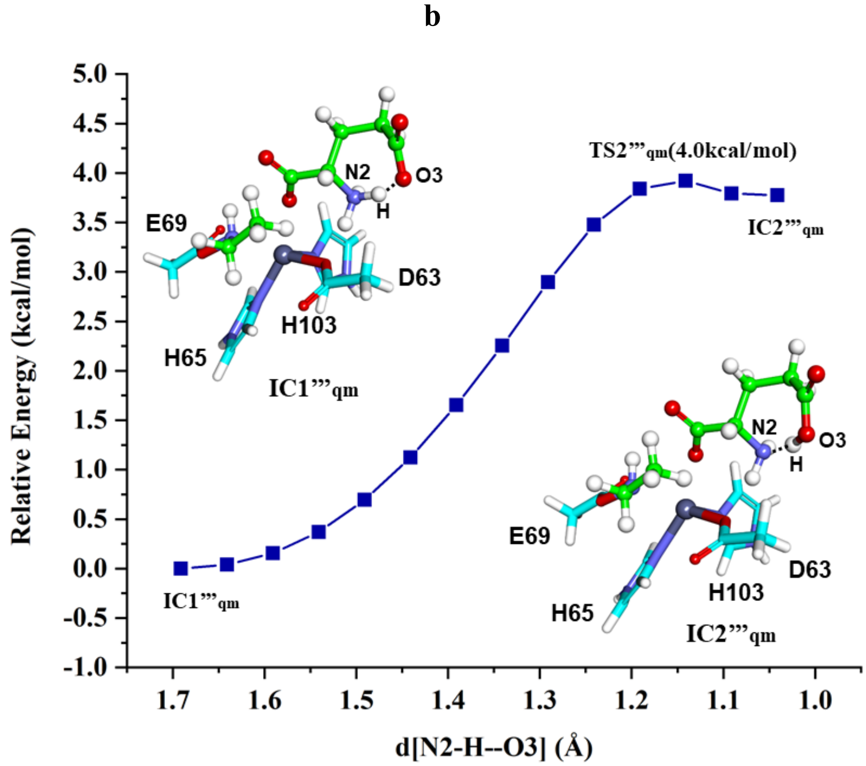

Supplementary Fig. 20 QM/MM (**a**) and QM (**b**) calculated energy profile for the proton transfer (PT) from the NH_3 to the carboxyl group within the substrate. The reaction coordinate is defined as the distance between H1 of N2-H_3 and O3 atom in substrate. **IC** = intermediate complex, **TS** = transition state.

Supplementary Fig. 21

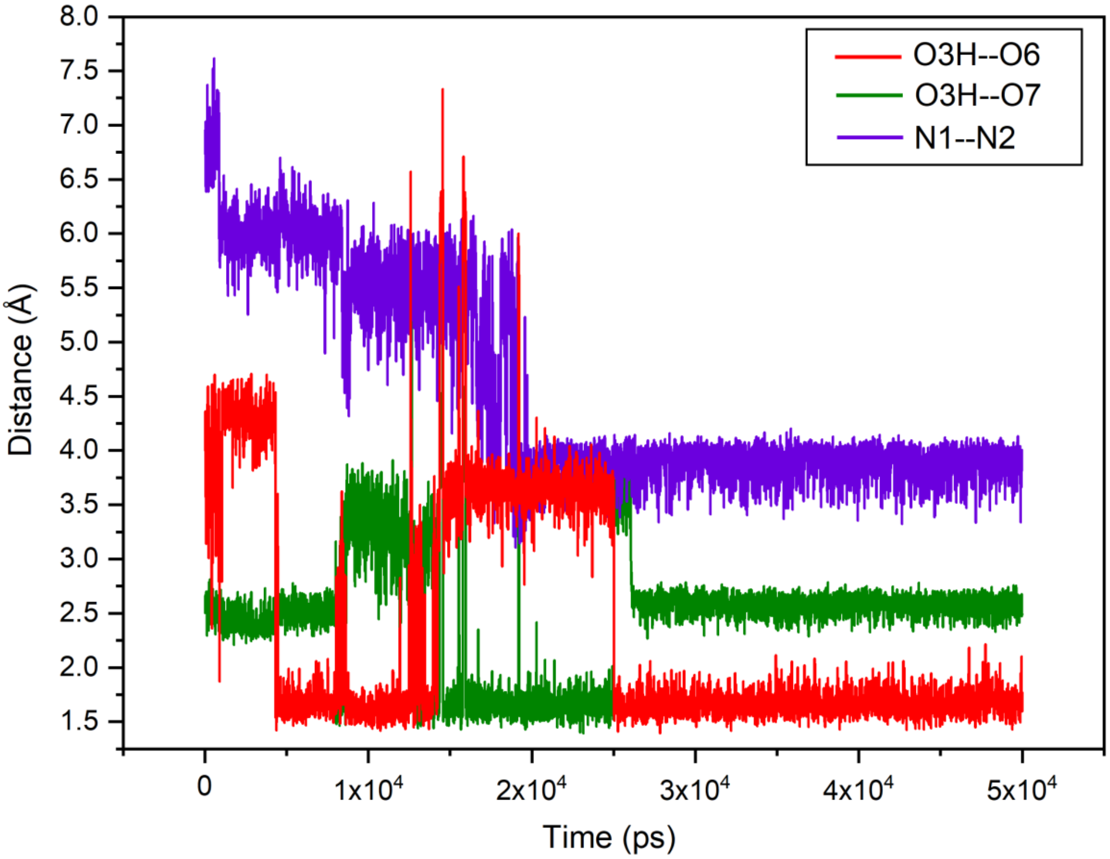

Supplementary Fig. 21 The fluctuation of key distances during the 50ns MD simulation of IC2. The distance between H atom in protonated carboxyl (-COO3-H group) and O6/O7 atoms in carboxyl group (-CO6O7 group) (red/green), N1 atom in imine group (-N1-H group) and N2 (-N2-H_2 group) atom in amino group (purple) during the MD simulation.

Supplementary Fig. 22

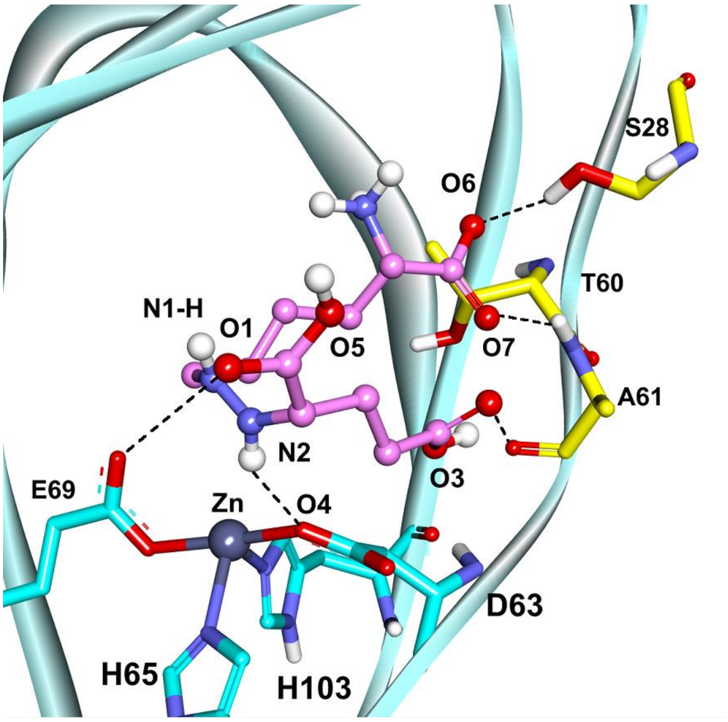

Supplementary Fig. 22 The docked conformation of product **1** in the zinc-binding cupin enzyme RHS1. The ball-and-stick model exposes docking conformations of substrate, the docked conformation of **1** is colored by pink and the key amino acid residues around zinc have been exhibited in cyan stick model

Supplementary Fig. 23

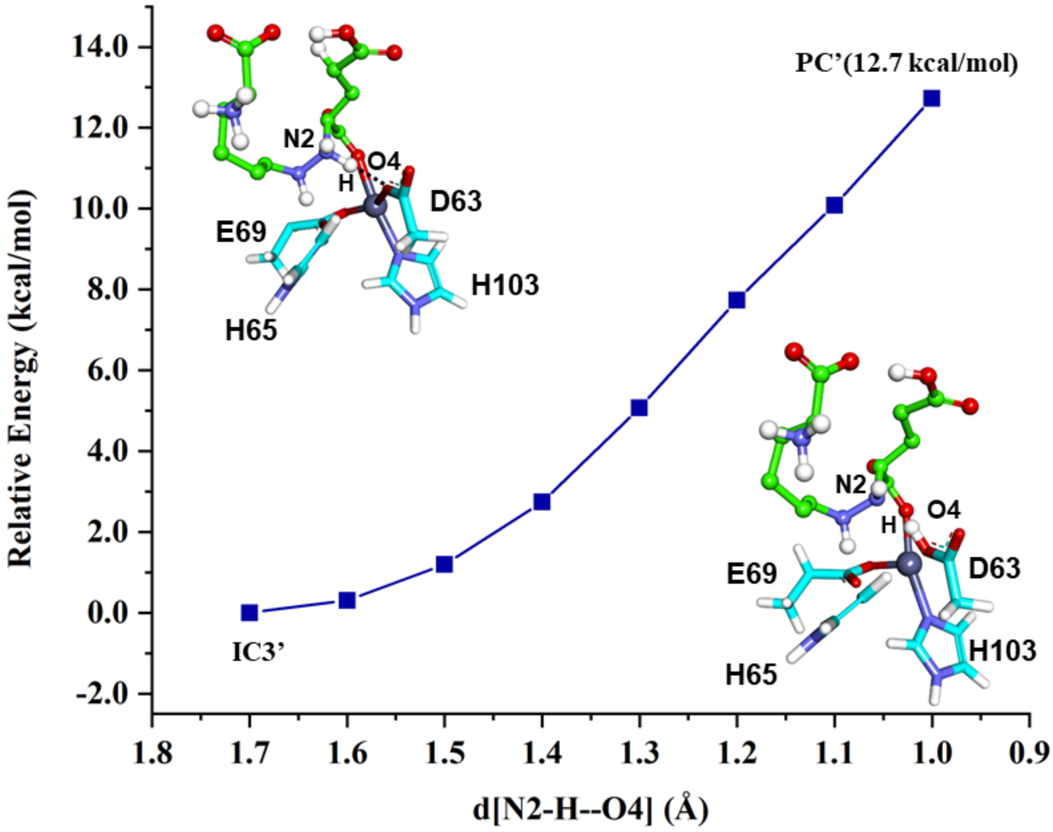

Supplementary Fig. 23 QM/MM calculated energy profile for the PT from the N2-H_2 to the carboxyl group of Asp63. The reaction coordinate is defined as the distance between H atom of N2-H_2 in substrate and O4 atom in Asp63. **IC** = intermediate complex, **PC** = product complex.

Supplementary Fig. 24

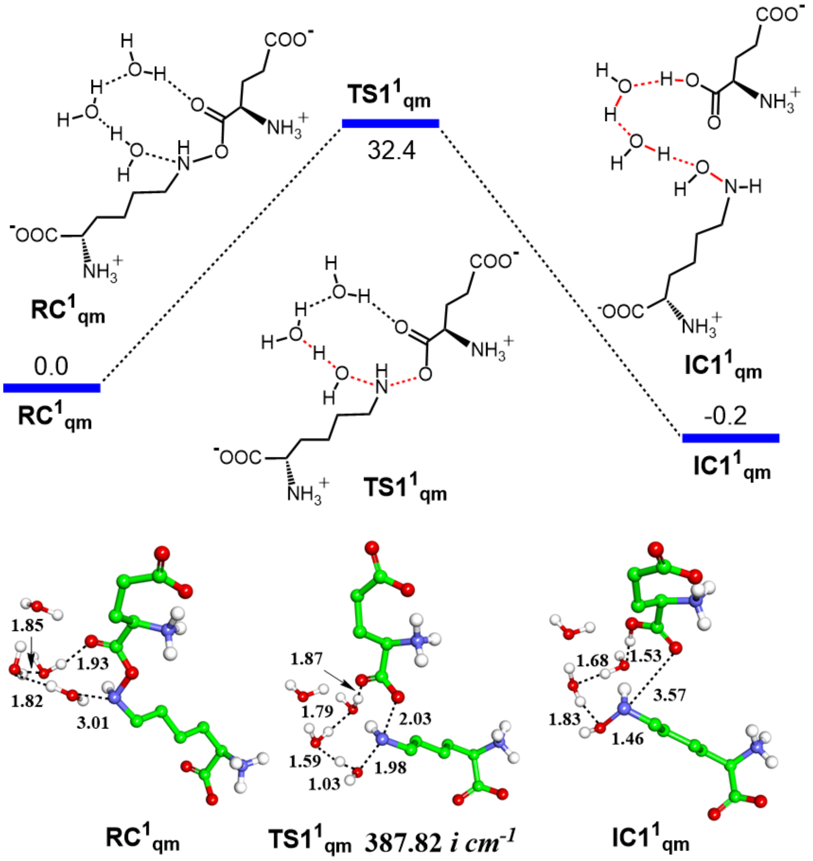

Supplementary Fig. 24 Cluster-continuum model calculated free energy profile (in kcal/mol) for the non-enzymatic N-O bond cleavage of ester substrate **4** at the BMK/6-311++G(d,p) level.

Supplementary Fig. 25

a

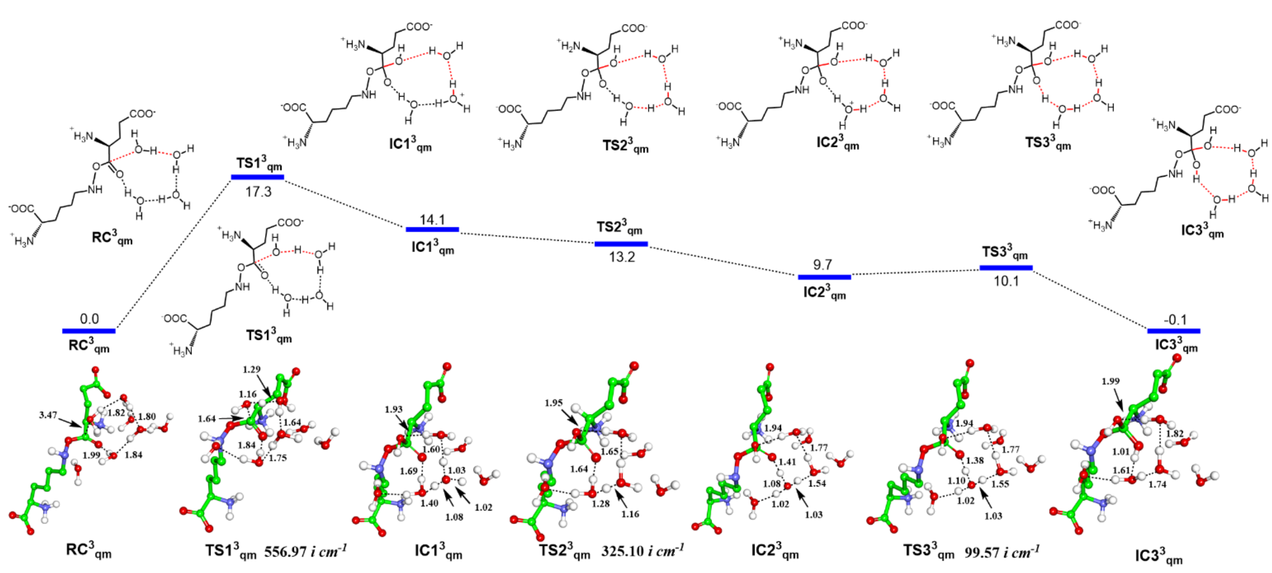

b

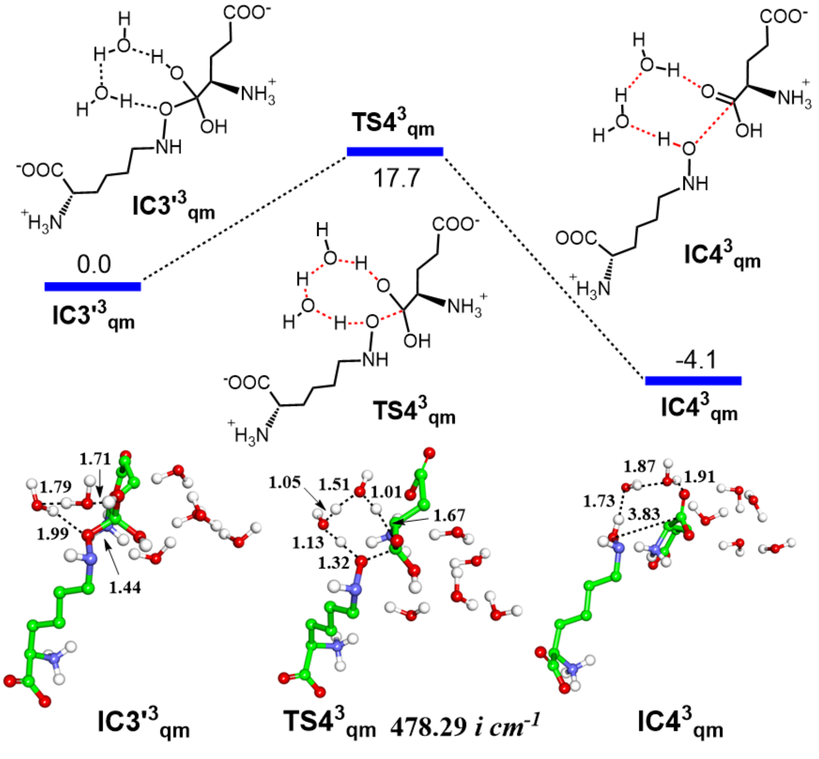

c

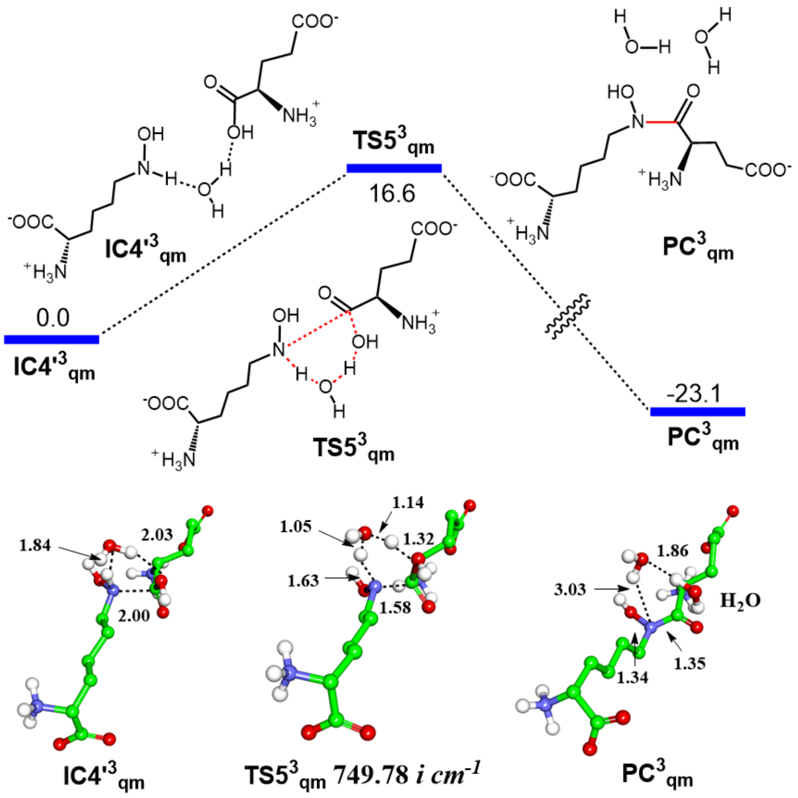

Supplementary Fig. 25 Investigation of the non-enzymatic reaction mechanism for the conversion of **4** to **3** (using the cluster-continuum model calculations) reveals that overall reaction involves three key steps (**a**-**c**). (**a**) Cluster-continuum model calculated free energy profile (in kcal/mol) for the hydration of ester substrate **4** (RC$^3_{qm}$ → IC3$^3_{qm}$) at the BMK/6-311++G(d,p) level. (**b**) Cluster-continuum model calculated free energy profile (in kcal/mol) for the C-O bond cleavage of IC3'$^3_{qm}$ at the BMK/6-311++G(d,p) level. (**c**) Cluster-continuum model calculated free energy profile (in kcal/mol) for the formation of amide product (3) at the BMK/6-311++G(d,p) level.

Overall, our above calculations show that the overall reaction involves three key steps. The first step (**a**) involves the hydration of carbonyl group, which requires an energy barrier of 17.3 kcal/mol, leading to hydration intermediate IC1$^3_{qm}$. The second step (**b**) involves the water-assisted proton transfer from the hydroxyl group to O atom (IC3'$^3_{qm}$ → TS4$^3_{qm}$, 17.7 kcal/mol), which could trigger the C-O cleavage to generate a hydroxylamine intermediate IC4$^3_{qm}$. The third step (**c**) involves the proton transfer from N-H group of hydroxylamine to OH group to generate the H_2O (IC4'$^3_{qm}$ → TS5$^3_{qm}$, 16.6 kcal/mol), which is coupled with C-O bond formation in the final amide product **3**.

Supplementary Fig. 26

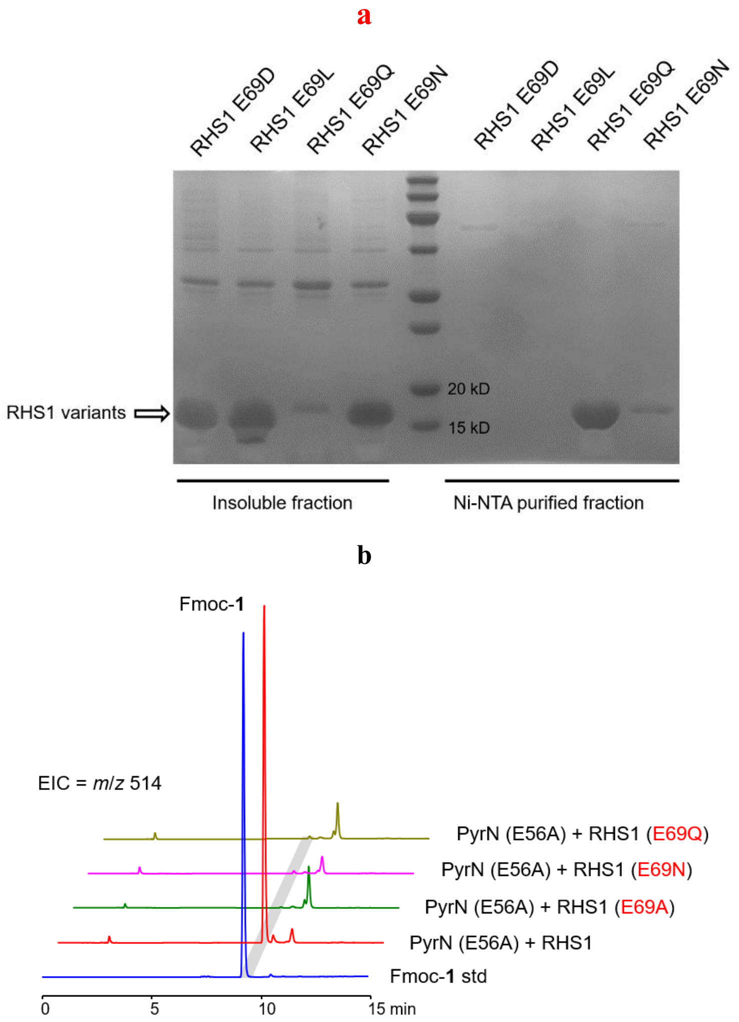

Supplementary Fig. 26 Characterization of RHS1 variants (E69D, E69N, E69Q and E69L). (**a**) SDS-PAGE analysis of the four RHS1 variants. Note: the experiment was repeated three times independently, and similar results were obtained. (**b**) LC-MS analysis (EIC=*m*/*z* 514, $[M+H]^+$ ion for Fmoc-**1**) of the *in vitro* reaction mixtures of RHS1 variants in the coupled reactions with PyrN (E56A), which provides **4** *in situ*.

Supplementary Fig. 27

a

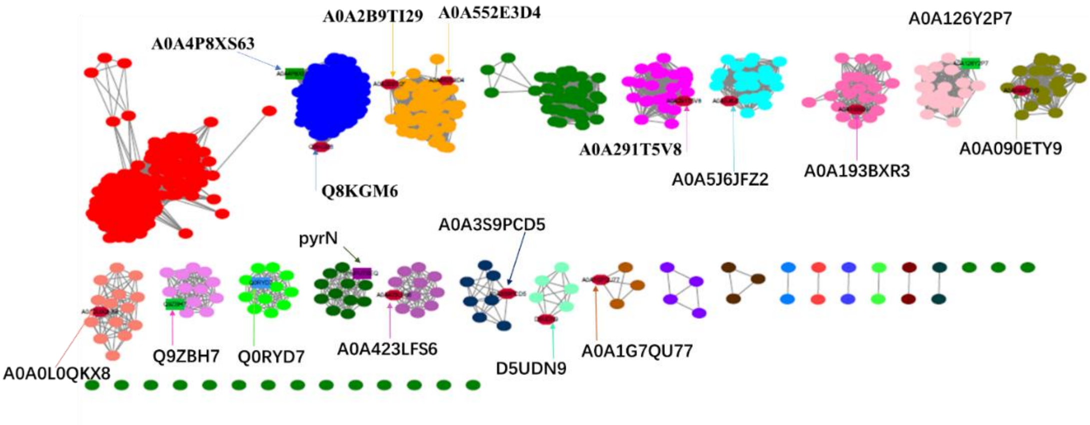

b

Uniprot ID	Size (aa)	Organism	Description
A0A4P8XS63	678	*Kitasatospora aureofaciens.*	Cupin-MetRS
Q8KGM6	684	*Rhizobium loti.*	Cupin-MetRS
A0A2B9TI29	671	*Bacillus cereus.*	Cupin-MetRS
A0A552E3D4	681	*Microcystis aeruginosa.*	Cupin-MetRS
A0A291T5Z9	125	*Streptomyces malaysiensis.*	Cupin
A0A291T5V8	529		MetRS
A0A5J6J753	142	*Streptomyces vinaceus.*	Cupin
A0A5J6JFZ2	547		MetRS
A0A193BXN9	122	*Amycolatopsis orientalis*	Cupin
A0A193BXR3	542		MetRS
A0A126Y2P7	650	*Streptomyces albidoflavus.*	Cupin-MetRS
A0A090ETY9	666	*Mesorhizobium plurifarium.*	Cupin-MetRS
A0A0L0QQM7	123	*Virgibacillus pantothenticus.*	Cupin
A0A0L0QKX8	552		MetRS
Q9ZBH8	128	*Streptomyces coelicolor.*	Cupin
Q9ZBH7	506		MetRS
Q0RYE0	122	*Rhodococcus jostii.*	Cupin
Q0RYD7	491		MetRS
A0A423LFS6	663	*Pseudomonas fluorescens.*	Cupin-MetRS
A0A3S9PCD2	116	*Streptomyces luteoverticillatus*	Cupin
A0A3S9PCD5	551		MetRS
D5UDN8	122	*Cellulomonas flavigena.*	Cupin
D5UDN9	513		MetRS
A0A1G7QU14	114	*Sinosporangium album.*	Cupin
A0A1G7QU77	550		MetRS

c

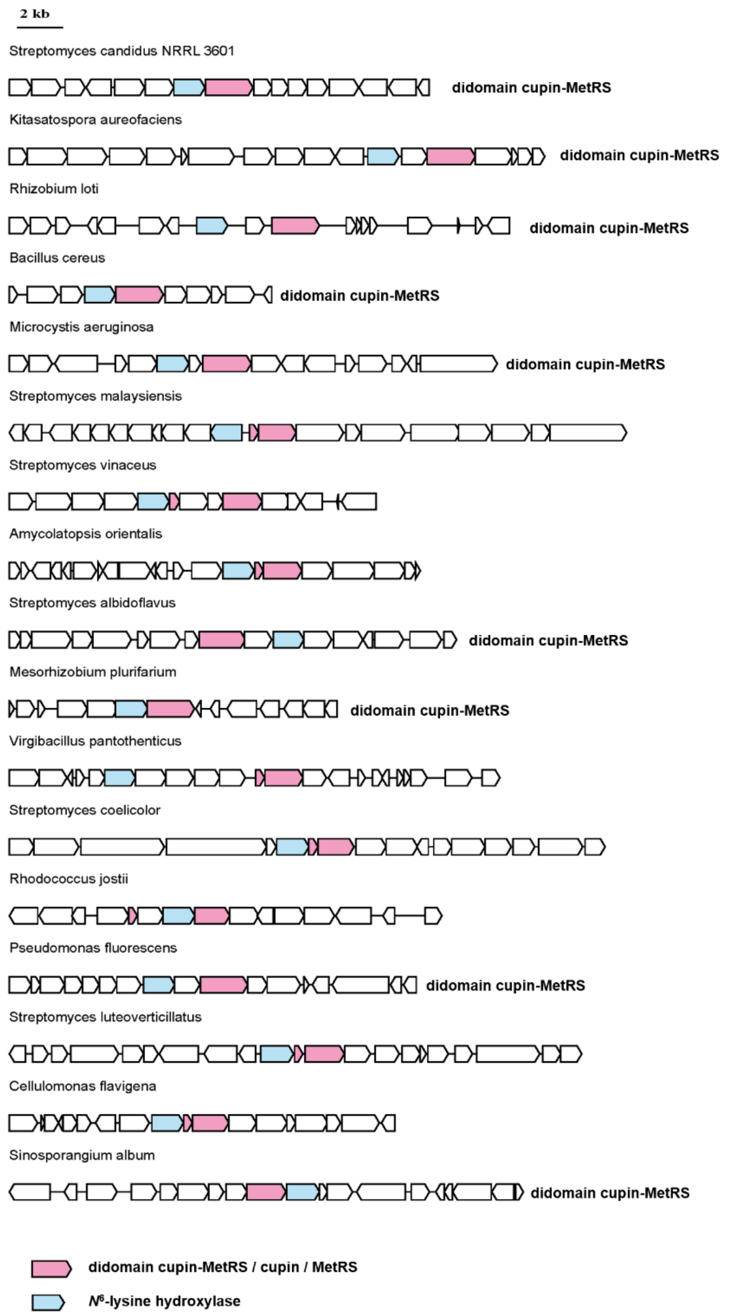

Supplementary Fig. 27. Database mining of PyrN homologues for substrate specificity study. (**a**) A sequence similarity network (SSN) of PyrN was generated using the EFI-Enzyme Similarity Tool with default settings. Edges connect sequences that align with an E-value threshold of 1.0 x 10^{-155}. The Uniprot ID of selected PyrN homologues targeted for gene synthesis, which are from different clusters in SSN, were indicated. (**b**) Detailed information of PyrN homologues targeted for gene synthesis and substrate specificity study. For cupin-MetRS di-domain proteins, the genes were directly synthesized. For standalone MetRS-like protein genes, its associated cupin protein genes were also synthesized, and these two genes were then co-expressed in the *E. coli* host. (**c**) Genomic contexts of the selected genes or gene pairs listed in (**b**). Note: all these genomic regions also contain N^6-lysine hydroxylase genes.

Supplementary Fig. 28

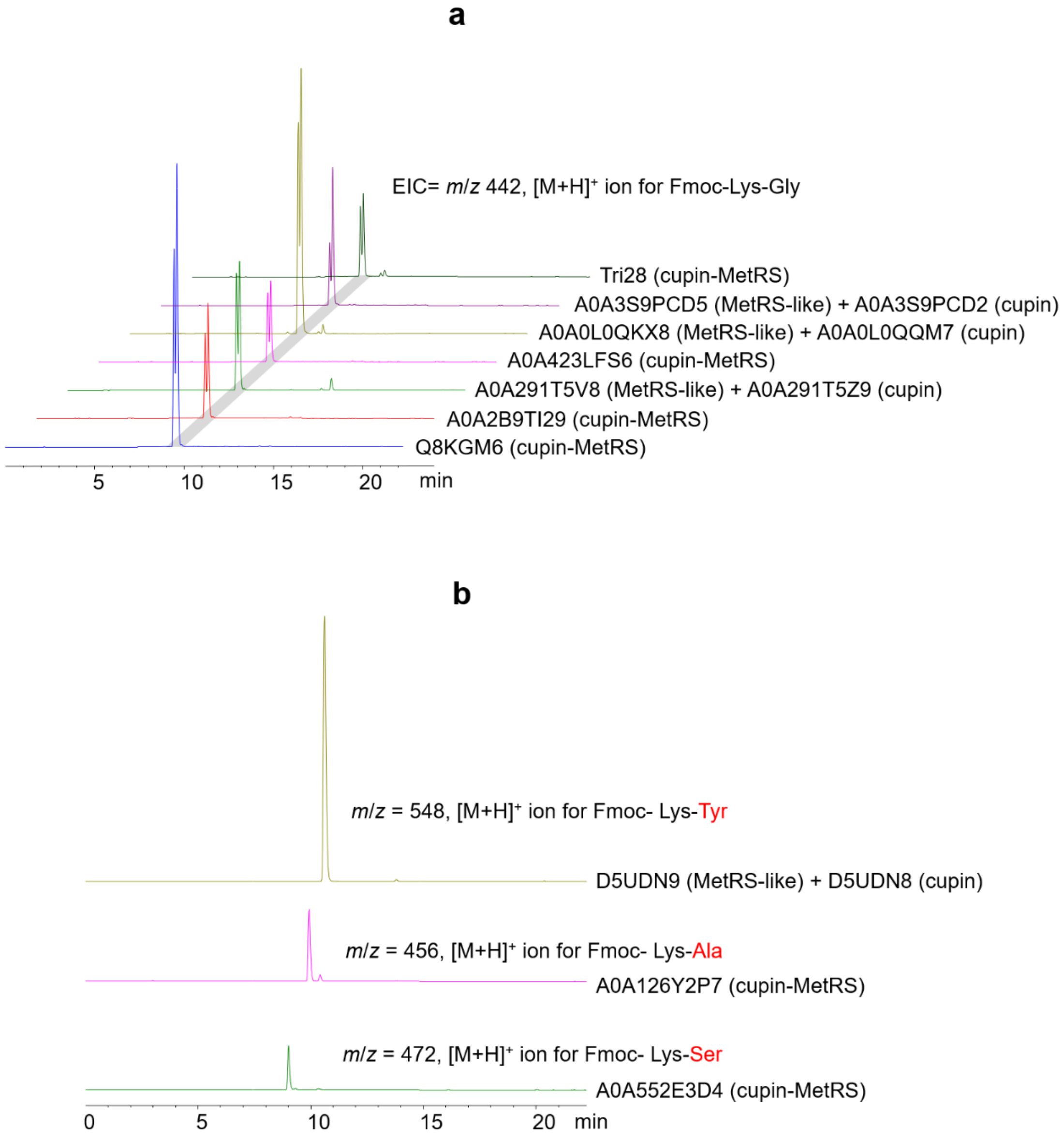

Supplementary Fig. 28 In vivo substrate specificity studies of PyrN homologues obtained by SSN-guided protein database mining. Selected genes were synthesized and introduced into *E. coli* host expressing N^6-lysine hydroxylase gene *nbtG*, and the culture supernatants of resulting *E. coli* strains were subjected to LC-MS analysis after Fmoc-Cl derivatization. (**a**) LC-MS analysis reveals genes or gene combinations that produce Lys-Gly conjugate in the above *E. coli in vivo* biotransformation assays. Tri28 from the triascins biosynthetic pathway were used as a positive control. (**b**) LC-MS analysis reveals genes or gene combinations that might produce new products (Lys-Tyr, Lys-Ala, Lys-Ser) in the biotransformation assays.

Supplementary Fig. 29

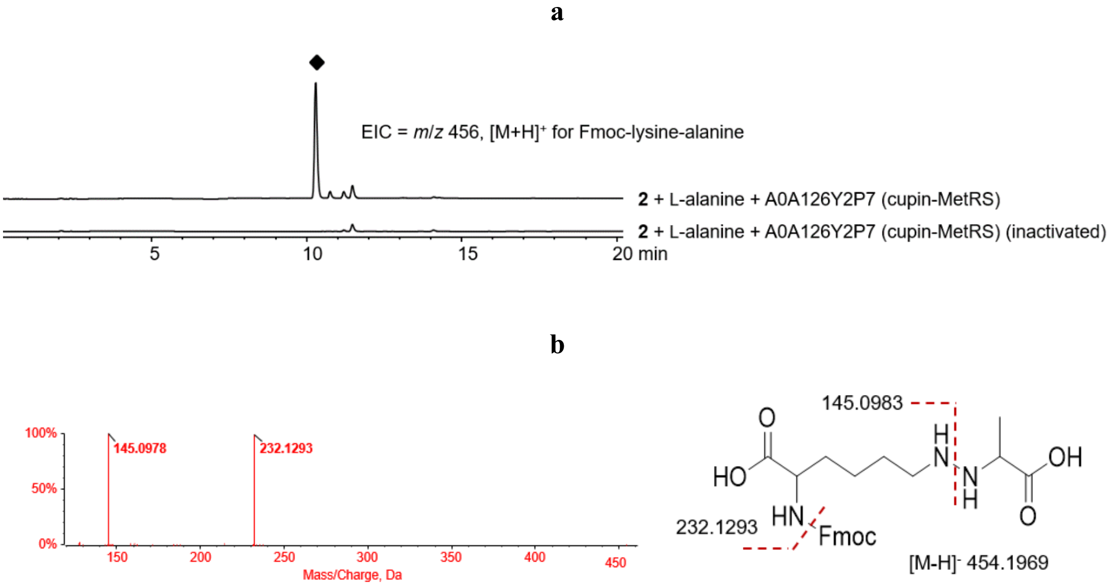

Supplementary Fig. 29 In vitro biochemical assay of enzyme (Uniprot accession number: A0A126Y2P7) that produces lysine-alanine conjugate. (**a**) LC-MS analysis of the reaction mixture of A0A126Y2P7 under positive detection mode. The product peak is indicated in black diamond. (**b**) LC-HR-MS/MS analysis of the product from (**a**) under negative detection mode. Note: we realize that we do not have the direct evidence about the N-N linkage, the structure of the product was suggested based on the fragment pattern and the protein sequence homology of this enzyme (A0A126Y2P7) to PyrN.

Supplementary Fig. 30

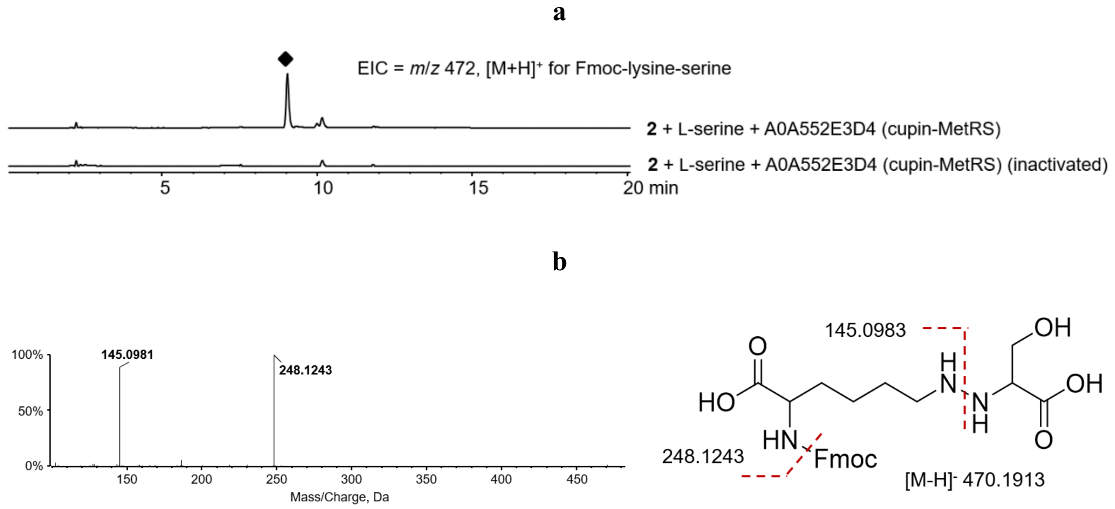

Supplementary Fig. 30 In vitro biochemical assay of enzyme (Uniprot accession number: A0A552E3D4) that produces lysine-serine conjugate. (**a**) LC-MS analysis of the reaction mixture of A0A552E3D4 under positive detection mode. The product peak is indicated in black diamond. (**b**) LC-HR-MS/MS analysis of the product from (**a**) under negative detection mode.

Supplementary Fig. 31

a

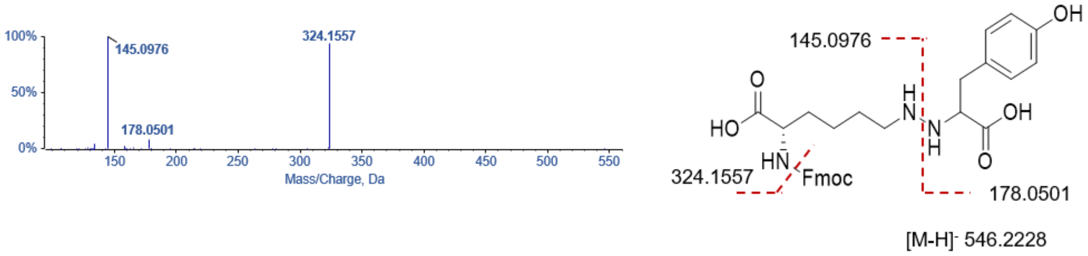

b

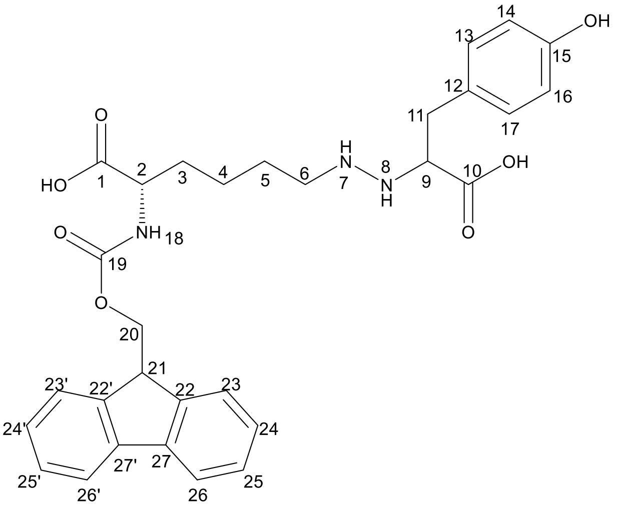

	NMR data of Lys-Tyr				
NO.	δ_H	δ_C	NO.	δ_H	δ_C
1	-	172.15	14,16	6.68 (2H,d, *J* =7.9Hz)	116.15
2	3.83 (1H,t, *J* = 6.3Hz)	53.96	15	-	157.05
3	1.74 (2H,brs)	31.13	19	-	158.48
4	1.17 (2H,brs)	22.90	20	4.56 (2H,d, *J* = 5.3Hz)	68.20
5	1.23 (2H,brs)	27.60	21	4.21 (1H,t, *J* = 5.3Hz)	48.45
6	2.94 (2H,brs)	50.66	22,22'	-	145.08
9	3.66 (1H,brs)	65.40	23,23'	7.55;7.58 (2H,d, *J*=7.4Hz)	125.68
10	-	176.45	24,24'	7.31 (2H,dt, *J* =7.5,3.8Hz)	128.17
11	2.80;2.61 (2H,brs)	37.66	25,25',	7.38 (2H,t, J =7.5Hz)	128.78
12	-	128.85	26,26'	7.78 (2H,d, J =7.5Hz)	120.91
13,17	6.97 (2H,brs)	131.27	27,27'	-	142.60

a ^{1}H (600 MHz) and ^{13}C (150 MHz) NMR Data in methanol-d4.

c

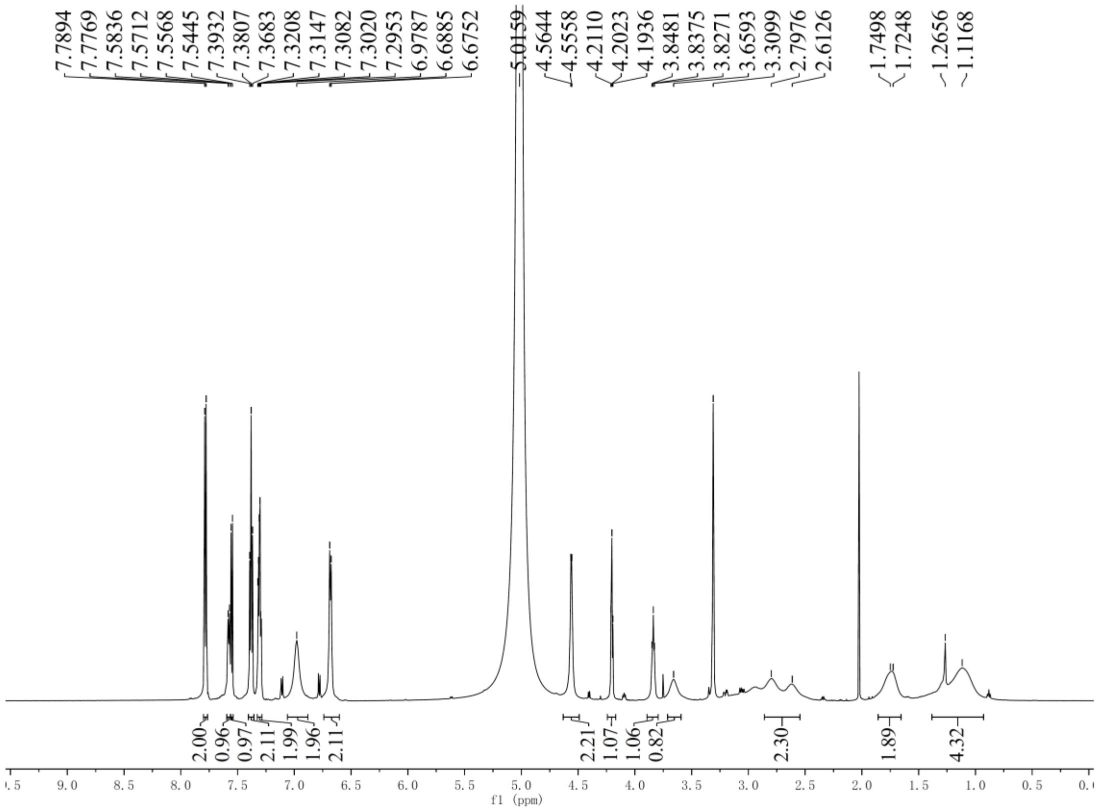
7.7894
7.7769
7.5836
7.5712
7.5568
7.5445
7.3932
7.3807
7.3683
7.3208
7.3147
7.3082
7.3020
7.2953
6.9787
6.6885
6.6752
5.0159
4.5644
4.5558
4.2110
4.2023
4.1936
3.8481
3.8375
3.8271
3.6593
3.3099
2.7976
2.6126
1.7498
1.7248
1.2656
1.1168
2.00
0.96
0.97
2.11
1.99
1.96
2.11
2.21
1.07
1.06
0.82
2.30
1.89
4.32
f1 (ppm)

d

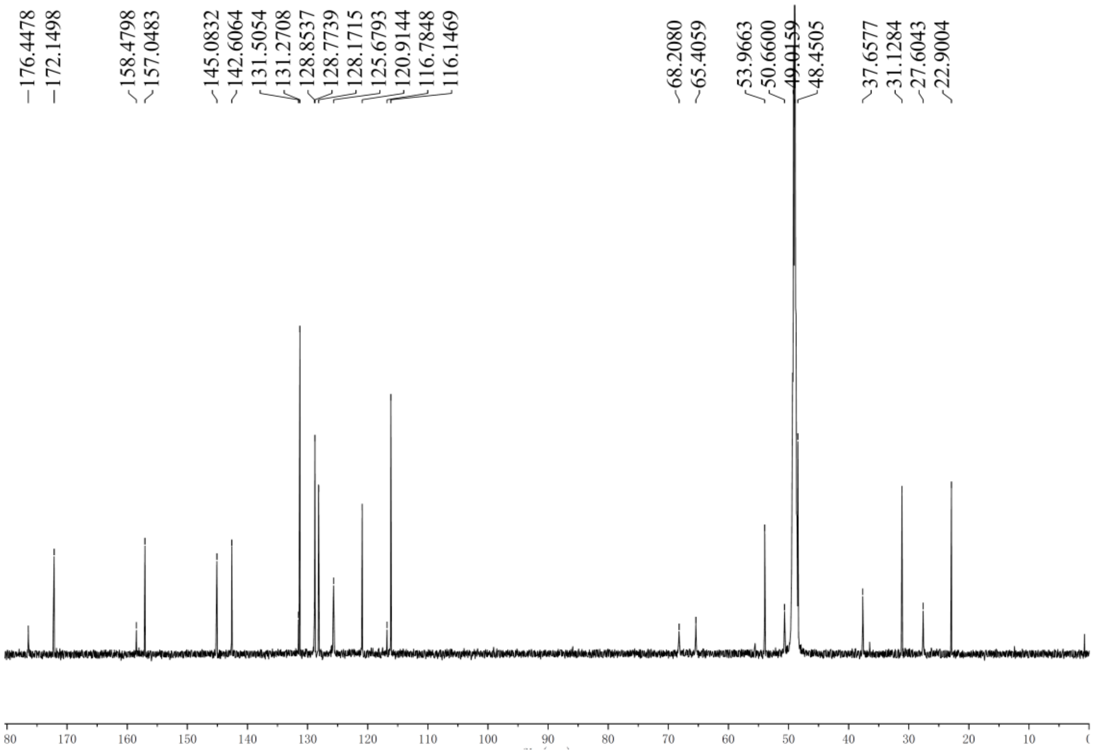
176.4478
172.1498
158.4798
157.0483
145.0832
142.6064
131.5054
131.2708
128.8537
128.7739
128.1715
125.6793
120.9144
116.7848
116.1469
68.2080
65.4059
53.9663
50.6600
49.0159
48.4505
37.6577
31.1284
27.6043
22.9004

e

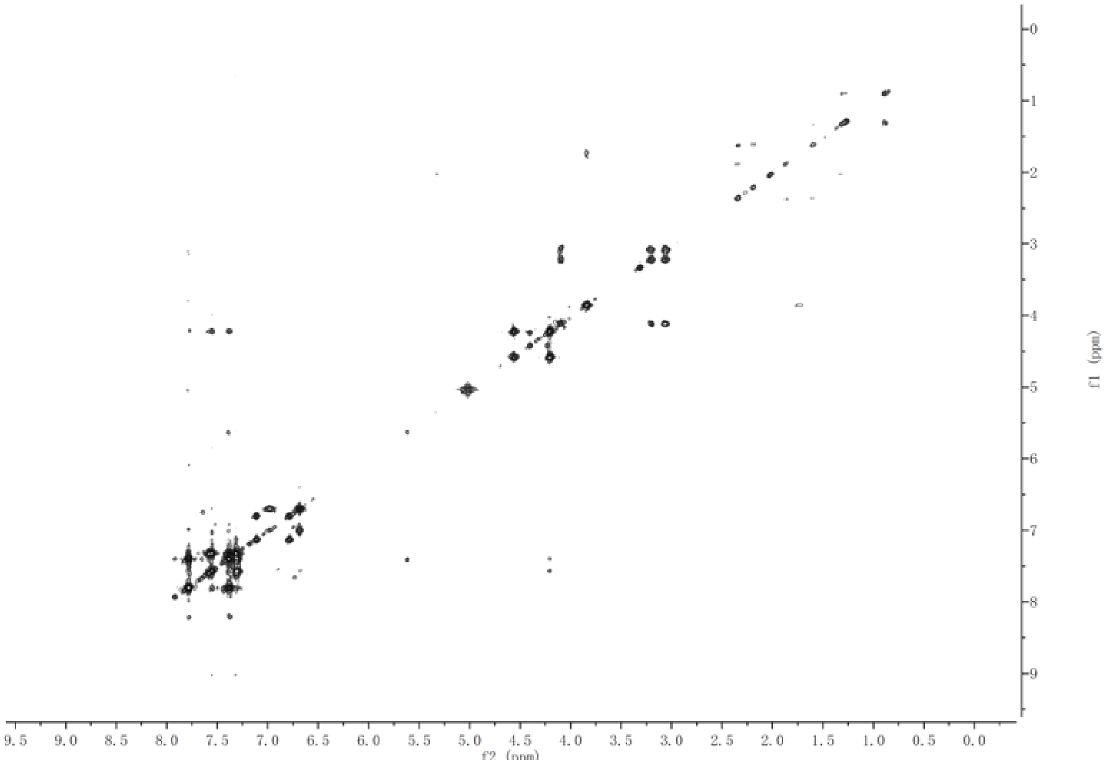

f

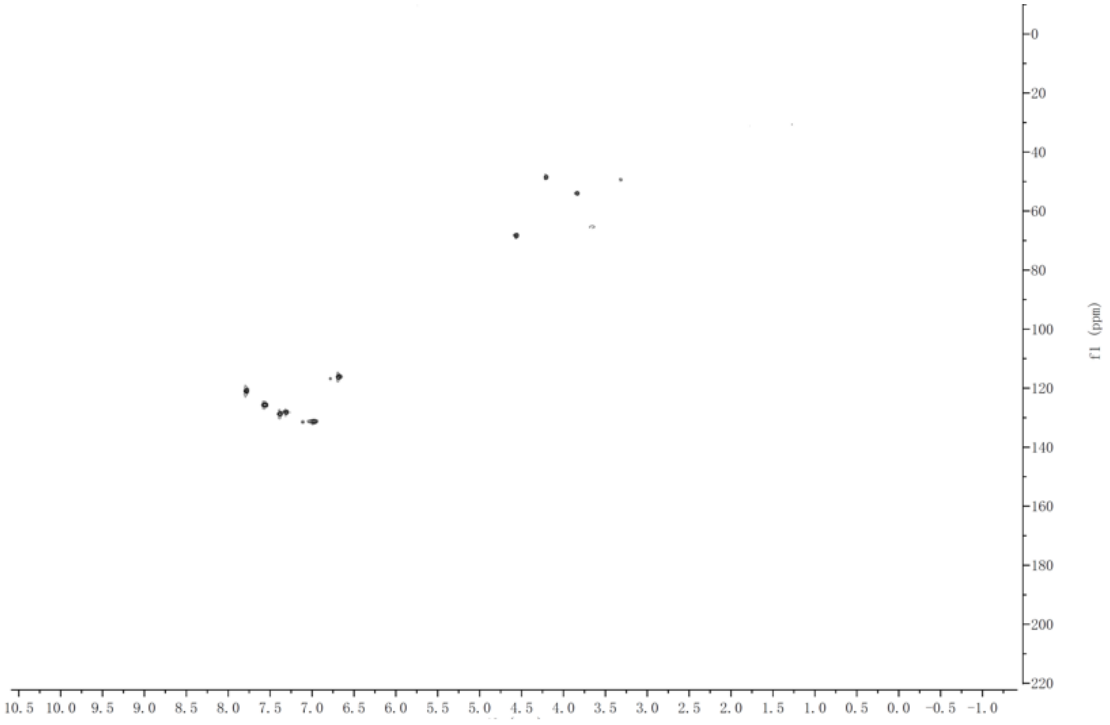

g

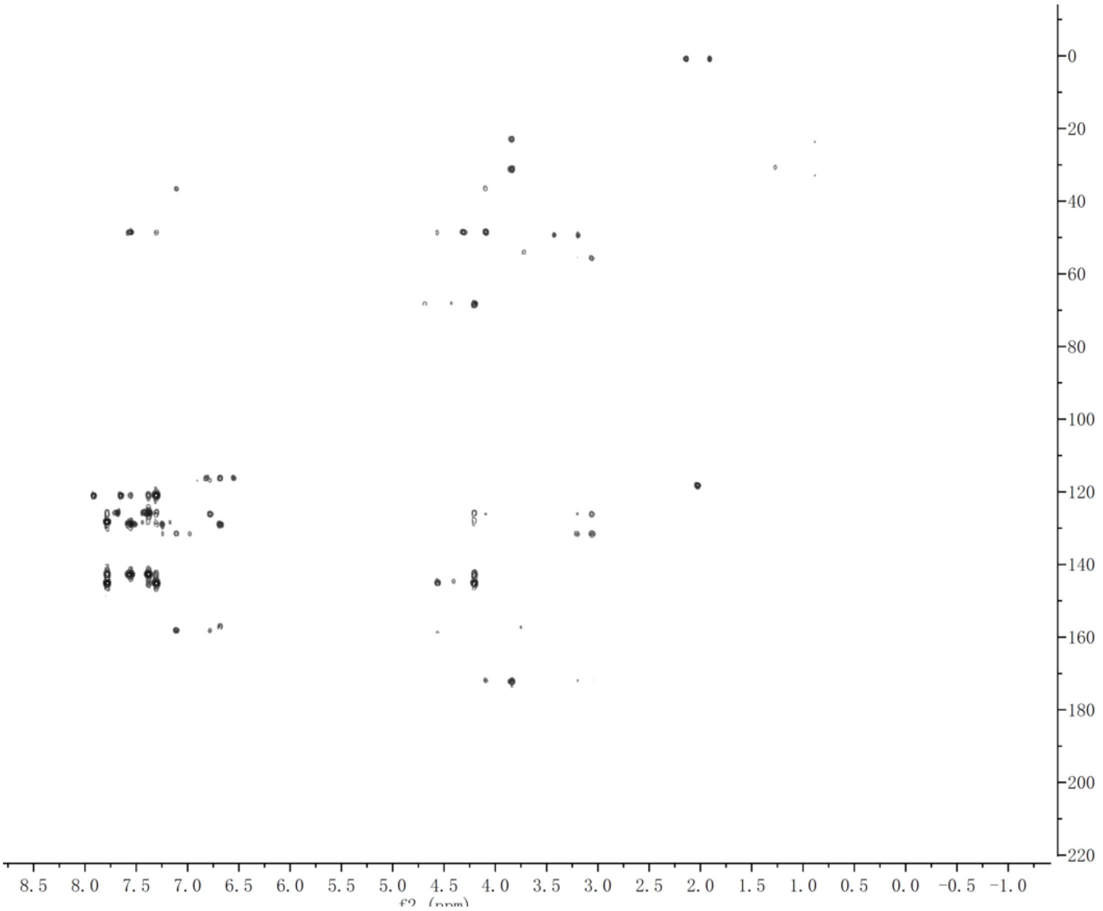

Supplementary Fig. 31 Characterization of the product lysine-tyrosine conjugate from enzyme pair D5UDN9 (MetRS-like) and D5UDN8 (cupin). Note: this product was isolated from the *in vivo* biotransformation assay using *E. coli* strain expressing *nbtG*, D5UDN9 gene, and D5UDN8 gene. For currently unknown reasons, we failed to reconstitute this reaction *in vitro*. (**a**) LC-HR-MS/MS analysis of the product lysine-tyrosine conjugate under negative detection mode. (**b**) NMR data for the isolated lysine-tyrosine conjugate. (**c**) ^{1}H NMR spectrum of lysine-tyrosine. (**d**) ^{13}C NMR spectrum of lysine-tyrosine. (**e**) ^{1}H-^{1}H COSY NMR spectrum of lysine-tyrosine. (**f**) HSQC NMR spectrum of lysine-tyrosine. (**g**) ^{1}H-^{13}C HMBC NMR spectrum of lysine-tyrosine.

Supplementary Fig. 32

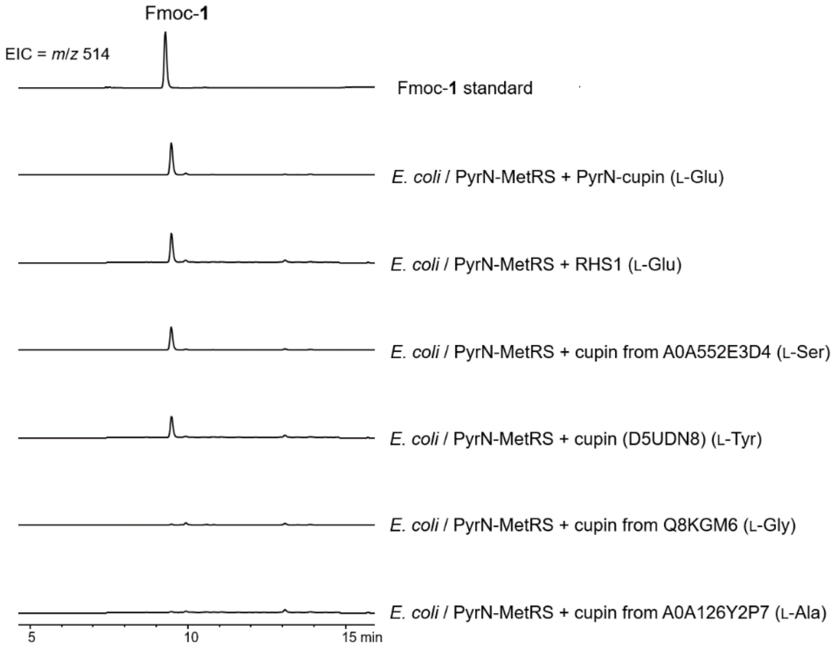

Supplementary Fig. 32 In vivo study of the substrate specificities of selected cupin domains/proteins in *E. coli* heterologous host expressing ^{6}N-lysine hydroxylase *nbtG*, PyrN-MetRS domain, and cupins. The results show that some domains/proteins have promiscuous substrate specificities, and the ones that naturally use amino acid with relatively larger side chains (L-tyrosine and L-serine) are also able to accept **4**.

Supplementary Fig. 33

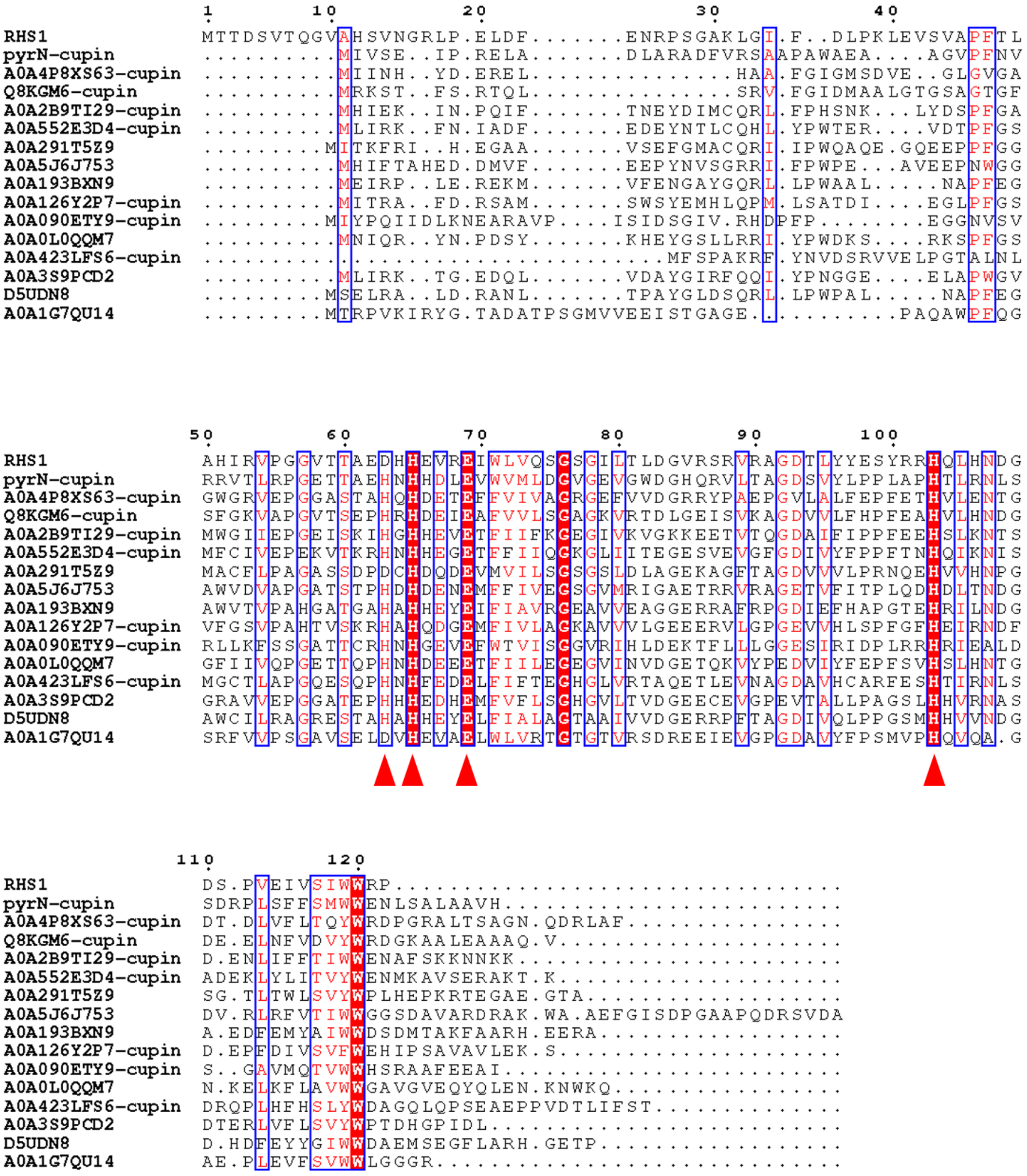

Supplementary Fig. 33 Sequence alignment of the cupin proteins/domains used in this study. Four putative metal-chelating residues (His/Asp, His, Glu, and His) are indicated with red triangles.
