# Supplementary material for: Molecular basis of enzymatic nitrogen-nitrogen formation by a family of zinc-binding cupin enzymes
Source: Nat Commun. 2021 Dec 10;12:7205. doi: 10.1038/s41467-021-27523-x (PMC8664883; doi:10.1038/s41467-021-27523-x)
Supplement: Supplementary file 3 — Reporting Summary [file 41467_2021_27523_MOESM3_ESM.pdf]

nature portfolio

Corresponding author(s): Yi-Ling Du

Last updated by author(s): Oct 27, 2021

# Reporting Summary

Nature Portfolio wishes to improve the reproducibility of the work that we publish. This form provides structure for consistency and transparency in reporting. For further information on Nature Portfolio policies, see our Editorial Policies and the Editorial Policy Checklist.

## Statistics

For all statistical analyses, confirm that the following items are present in the figure legend, table legend, main text, or Methods section.

| n/a | Confirmed | |
|---|---|---|
| ☐ | ☒ | The exact sample size ($n$) for each experimental group/condition, given as a discrete number and unit of measurement |
| ☐ | ☒ | A statement on whether measurements were taken from distinct samples or whether the same sample was measured repeatedly |
| ☒ | ☐ | The statistical test(s) used AND whether they are one- or two-sided<br>*Only common tests should be described solely by name; describe more complex techniques in the Methods section.* |
| ☒ | ☐ | A description of all covariates tested |
| ☒ | ☐ | A description of any assumptions or corrections, such as tests of normality and adjustment for multiple comparisons |
| ☐ | ☒ | A full description of the statistical parameters including central tendency (e.g. means) or other basic estimates (e.g. regression coefficient) AND variation (e.g. standard deviation) or associated estimates of uncertainty (e.g. confidence intervals) |
| ☒ | ☐ | For null hypothesis testing, the test statistic (e.g. $F$, $t$, $r$) with confidence intervals, effect sizes, degrees of freedom and $P$ value noted<br>*Give P values as exact values whenever suitable.* |
| ☒ | ☐ | For Bayesian analysis, information on the choice of priors and Markov chain Monte Carlo settings |
| ☒ | ☐ | For hierarchical and complex designs, identification of the appropriate level for tests and full reporting of outcomes |
| ☒ | ☐ | Estimates of effect sizes (e.g. Cohen's $d$, Pearson's $r$), indicating how they were calculated |

*Our web collection on statistics for biologists contains articles on many of the points above.*

## Software and code

Policy information about availability of computer code

| | |
|---|---|
| Data collection | The pka value of residues was predicted by PROPKA ( https://server.poissonboltzmann.org/pdb2pqr). Molecular docking was performed with AutoDock Vina tool 1.2.0 in Chimera UCSF. MD simulations were performed with Amber18. QM/MM was performed with ChemShell 3.7.0, combining turbomole 2018 for the QM region. Gaussian 16 was adopted for the optimization and frequencies calculation of models relative to the catalytic processes. |
| Data analysis | We used OriginPro learning edition (https://www.originlab.com/OriginProLearning.aspx) for the analysis of MD and QM data. |

For manuscripts utilizing custom algorithms or software that are central to the research but not yet described in published literature, software must be made available to editors and reviewers. We strongly encourage code deposition in a community repository (e.g. GitHub). See the Nature Portfolio guidelines for submitting code & software for further information.

## Data

Policy information about availability of data

All manuscripts must include a data availability statement. This statement should provide the following information, where applicable:

- Accession codes, unique identifiers, or web links for publicly available datasets
- A description of any restrictions on data availability
- For clinical datasets or third party data, please ensure that the statement adheres to our policy

The data generated in this study are provided in this Article, Supplementary Information and Source data files (the source data underlying Supplementary Figs 1, 3c, 13a, and 26a are provided as a Source Data file). All other relevant data are available from the corresponding author upon request.

# Field-specific reporting

Please select the one below that is the best fit for your research. If you are not sure, read the appropriate sections before making your selection.

- [x] Life sciences
- [ ] Behavioural & social sciences
- [ ] Ecological, evolutionary & environmental sciences

For a reference copy of the document with all sections, see nature.com/documents/nr-reporting-summary-flat.pdf

# Life sciences study design

All studies must disclose on these points even when the disclosure is negative.

| | |
|---|---|
| Sample size | Each experiment in this study was repeated independently at least three times. These sample sizes were chosen based on convention in the field. |
| Data exclusions | No data were excluded. |
| Replication | Measurements were repeated at least three times and all were reproducible. |
| Randomization | Randomization was not required for any of the experiments in this study. |
| Blinding | Blinding was not required for any of the experiments in this study. |

# Reporting for specific materials, systems and methods

We require information from authors about some types of materials, experimental systems and methods used in many studies. Here, indicate whether each material, system or method listed is relevant to your study. If you are not sure if a list item applies to your research, read the appropriate section before selecting a response.

## Materials & experimental systems

| n/a | Involved in the study | |
|---|---|---|
| [x] | [ ] | Antibodies |
| [x] | [ ] | Eukaryotic cell lines |
| [x] | [ ] | Palaeontology and archaeology |
| [x] | [ ] | Animals and other organisms |
| [x] | [ ] | Human research participants |
| [x] | [ ] | Clinical data |
| [x] | [ ] | Dual use research of concern |

## Methods

| n/a | Involved in the study | |
|---|---|---|
| [x] | [ ] | ChIP-seq |
| [x] | [ ] | Flow cytometry |
| [x] | [ ] | MRI-based neuroimaging |
